# Global trends and hot topics in electrical stimulation of skeletal muscle research over the past decade: A bibliometric analysis

**DOI:** 10.3389/fneur.2022.991099

**Published:** 2022-10-05

**Authors:** Yi Huang, Yuxiang Gong, Yu Liu, Jianqiang Lu

**Affiliations:** Key Laboratory of Exercise and Health Sciences of Ministry of Education, Shanghai University of Sport, Shanghai, China

**Keywords:** skeletal muscle, electrical stimulation, bibliometric analysis, visualization, network analysis

## Abstract

**Background:**

Over the past decade, numerous advances have been made in the research on electrical stimulation of skeletal muscle. However, the developing status and future direction of this field remain unclear. This study aims to visualize the evolution and summarize global research hot topics and trends based on quantitative and qualitative evidence from bibliometrics.

**Methods:**

Literature search was based on the Web of Science Core Collection (WoSCC) database from 2011 to 2021. CiteSpace and VOSviewer, typical bibliometric tools, were used to perform analysis and visualization.

**Results:**

A total of 3,059 documents were identified. The number of literature is on the rise in general. Worldwide, researchers come primarily from North America and Europe, represented by the USA, France, Switzerland, and Canada. The Udice French Research Universities is the most published affiliation. Millet GY and Maffiuletti NA are the most prolific and the most co-cited authors, respectively. *Plos One* is the most popular journal, and the *Journal of Applied Physiology* is the top co-cited journal. The main keywords are muscle fatigue, neuromuscular electrical stimulation, spinal cord injury, tissue engineering, and atrophy. Moreover, this study systematically described the hotspots in this field.

**Conclusion:**

As the first bibliometric analysis of electrical stimulation of skeletal muscle research over the past decade, this study can help scholars recognize hot topics and trends and provide a reference for further exploration in this field.

## Introduction

Skeletal muscle is the center of contractile force production and has a great capacity for regeneration (1–3). However, the loss of muscle functionality is unavoidable under exceptional circumstances, such as serious injury. In the entire lifespan, maintaining skeletal muscle mass and function is crucial for preserving metabolic health (4) and supporting independent locomotion. Limb muscle dysfunction often occurs as a comorbidity of many diseases (5, 6) and has significant clinical implications like reduction of exercise tolerance and quality of life, even survival. Although exercise training may be a potent intervention to resolve the situation (7, 8), other therapy, such as neuromuscular electrical stimulation (NMES) (9, 10), is widely used as well. In addition to the health issue, optimal strategies for skeletal muscle remodeling (11) and reconditioning are also noteworthy. Particularly within the athletic community, there is a great interest in enhancing skeletal muscle adaptation to training (12) to maximize physical potential in competitive events.

Since the first observation of the peroneal nerve stimulated by electric current to correct foot drop in hemiplegic patients (13), many studies have demonstrated electrical stimulation's therapeutic effects on neuromuscular disorders (14–16). A variety of new stimulation patterns and techniques have been explored. With the development of brain-behavioral relationships and pathophysiological aspects of various neurological disorders, invasive and non-invasive stimulation has been applied experimentally and clinically (17–19). In addition, there needs to be more evidence of optimizing the electrical stimulation effect, such as selecting efficient parameters and updating appropriate biological electrode materials.

Over the past few decades, electropathy of skeletal muscle has made significant progress. Ethier et al. performed functional electrical stimulation (FES) by implanted microelectrodes to restore voluntary movement in paralyzed patients (20). With continuous technological innovation, more and more literature has emerged on the electrical stimulation of skeletal muscle research, but the current research status is unclear.

Bibliometric analysis, a popular and rigorous method, is used for evaluating and exploring large volumes of published scientific output. It follows specific techniques and procedures and is based on qualitative and quantitative evidence (21). Bibliometrics can reveal collaboration patterns, research constituents, and emerging trends, as well as explore the knowledge structure of a given field, thus helping researchers to have a quick overview of a research area, identify gaps and obtain novel ideas (22). In recent years, bibliometric analysis has gained in popularity in medical research, and this phenomenon can be due to (1) the availability and advancement of analysis software like VOSviewer, CiteSpace, Leximancer, and scientific databases like Web of Science, and (2) the intercrossed application of the bibliometric methodology from information science to medical research. Previous bibliometric analysis of electrical stimulation research involves pelvic floor physiotherapy (23) and invasive and non-invasive brain stimulation (24–26). The developing status and prospects of the research on electrical stimulation of skeletal muscle are knowledge gaps in the current medical literature.

Therefore, we performed a bibliometric analysis of electrical stimulation of skeletal muscle research over the past decade (2011–2021). This study aims to visualize the evolution and summarize the hot topics and trends in this research field. Furthermore, the emphases and prospects of subsequent research are suggested.

## Materials and methods

### The techniques of bibliometric analysis

Performance analysis and science mapping are two core techniques of bibliometric analysis. Essentially, performance analysis focuses on the contributions of different research constituents (such as countries, institutions, authors, and journals), and science mapping accounts for the relationships between them (27).

Performance analysis, which is descriptive, is standard practice for bibliometric studies. The most significant measures of the analysis are the publications quantities and citations (annual or per research constituent). Specifically, publications reflect productivity, while citations provide an estimate of influence.

Science mapping techniques include the analysis of citation, co-citation, co-authorship, and co-word. These techniques, especially in combination with network analysis, help researchers understand the intellectual linkages between research constituents and the knowledge structure of the research field. Table 1 summarizes several different scientific mapping techniques.

**Table 1:** Techniques for science mapping.

**Category**	**Data types**	**Unit of analysis**	**Function**
Citation analysis	Author name, citations, title, journals, references	Documents	Identifying the most influential publications
Co-citation analysis	References	Documents	Understanding the development of the foundational themes
Co-authorship analysis	Author, countries, institutions	Documents	Examining the social interactions or intellectual relationships between research constituents
Co-word analysis	Title, abstract, author keywords	Words	Exploring the existing or future relationships among research topics

Citation is the most objective and direct indicator to evaluate the impact of a publication (28). In citation analysis, the number of citations a paper receives determines its impact. Therefore, we can ascertain the most influential publications and thus understand the intellectual dynamics of a research field.

Co-citation analysis assumes that publications cited together frequently are similar in theme (29). The connections among co-occur publications form a co-citation network. Researchers use co-citation analysis to identify authoritative publications and excavate thematic clusters. Co-cited is defined as authors, journals, or references cited together by researchers.

Co-authorship analysis reveals the intellectual collaboration among researchers, countries, and institutions (30). Since it exposes the dominant authors and regions, co-authorship analysis provides a potential opportunity for prospective scholars to contact and cooperate with influential and trending scholars in their research field. In addition, this analysis maps collaborative relationships across periods, allowing scholars to review the intellectual development trajectory underlying collaboration networks.

Co-word analysis examines the thematic relationships between the words that frequently appear together. The terms analyzed usually come from author keywords but can also be derived from titles, abstracts, and full texts. Co-word analysis can present representative content of each thematic cluster and can be used to predict future directions in a research field.

### Data source and search strategy

**Figure 1:**
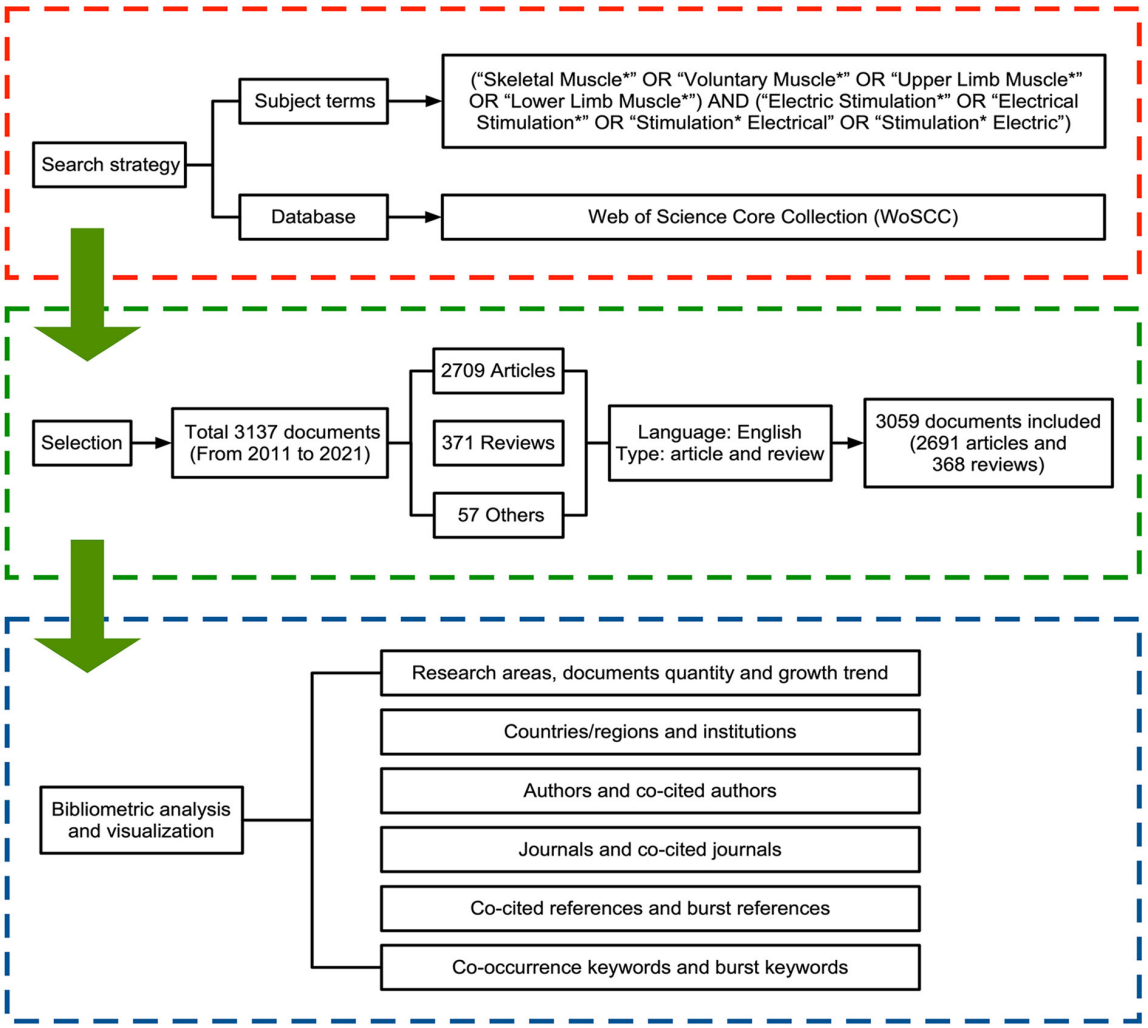
Flow chart of bibliometric research.

Comprehensive publications retrieved were based on the Web of Science Core Collection (WoSCC), and the MeSH major topics query confirmed subject terms (31, 32). The first data collection was completed on June 8, 2022, and updated on September 8, 2022, by searching the WoSCC for literature published between 2011 and 2021. The subject terms were as follows: (“Skeletal Muscle^*^” OR “Voluntary Muscle^*^” OR “Upper Limb Muscle^*^” OR “Lower Limb Muscle^*^”) AND (“Electric Stimulation^*^” OR “Electrical Stimulation^*^” OR “Stimulation^*^ Electrical” OR “Stimulation^*^ Electric”). Only research articles and reviews were included, and the language was restricted to English. A total of 3,059 records were extracted in this study. Figure 1 shows the retrieval process and research framework.

Since this study did not involve animal or human experiments and all data were obtained from the open database, an ethical statement was not required.

### Data analysis

The retrieved data were processed directly using the WoSCC built-in analysis module and then exported in bibliometrics software for further analysis. WoSCC enables the export of primary data, such as document amounts, citation reports, authors' information, countries/regions, affiliations, and journals. MATLAB R2020a was used to draw relevant figures.

VOSviewer and CiteSpace software, visual and synthetic analytic systems, were selected for data mining and mapping the retrieved articles (33–36).

The VOSviewer (version 1.6.18) was used to integrate primary information about co-citation on authors, collaborations between countries or institutions, and co-occurrence on keywords into network maps. It is obvious to judge the importance through the node's size and the line's thickness in the classified colored network maps. Large circles and labels signify great weights; broad lines signify strong relationships. Different colors can distinguish clusters. Then hotspots and trends in electrical stimulation of skeletal muscle studies are visualized in the co-word network.

The CiteSpace (version 6.1.R2) was used for authors, institutions, highest citation journals and references, and burst detection. Centrality reflects the significance of a node in the network. The citation burst demonstrates a keyword or a reference frequently occurring in a specific period (37). Potential research frontiers can be dug out *via* burst keywords and references.

## Results

### Overview of published documents

A total of 3,137 records were identified. The main types were articles (86.35%) and reviews (11.83%), accounting for almost all publications. Therefore, we concentrated on analyzing and assessing these two types of papers (a total of 3,059).

**Figure 2:**
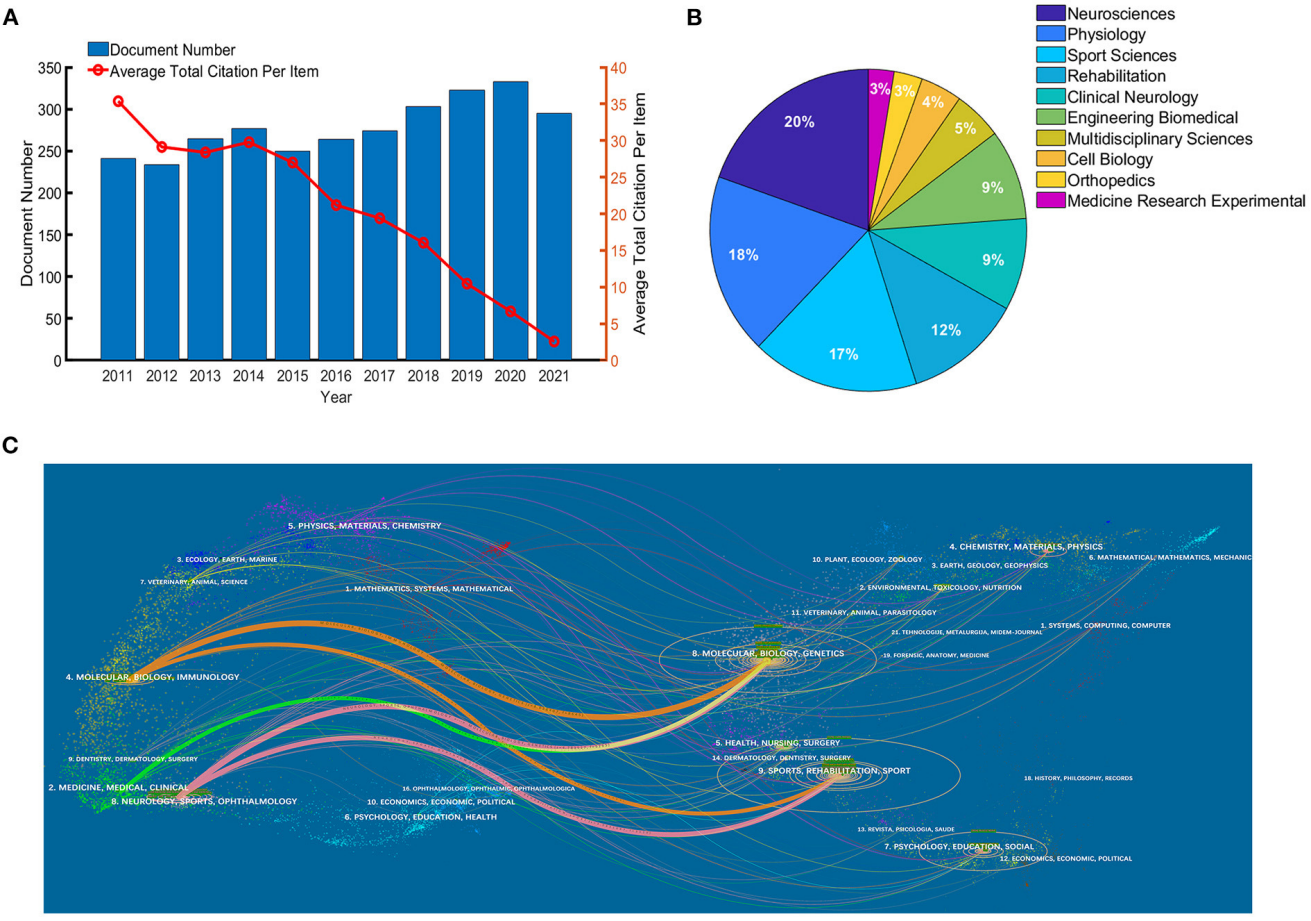
**(A)** Growth trend in publications from 2011 to 2021. **(B)** Research areas of publications. **(C)** Dual-map overlay of journals.

Figure 2A shows the number of publications on electrical stimulation of skeletal muscle research and the average total citations per paper (the red line). During the last 10 years, the literature number fluctuated but generally showed an upward trend, and the average total citations per literature were kept at least 10 times (from 2011 to 2019). The yearly output exceeded 300 in 2018. However, the volume of papers was down in 2021.

Furthermore, we performed an analysis of research areas and disciplines. Neurosciences is the most published area of study, followed by Physiology, Sport Sciences, and Rehabilitation, as shown in Figure 2B. A dual-map overlay was structured in Figure 2C to refine the research basis further. The colored paths in the dual-map overlay represent the citation relationship of both sides. On the left is the journal distribution of citing references, science mapping such as molecular, biology, medicine, clinical, neurology, and sports, representing the primary discipline and could be regarded as the application fields. On the right side is the cited references distribution, like molecular, biology, genetics, sport, and rehabilitation, signifying the disciplines mainly referenced and research basis.

### Leading countries/regions and affiliations

**Figure 3:**
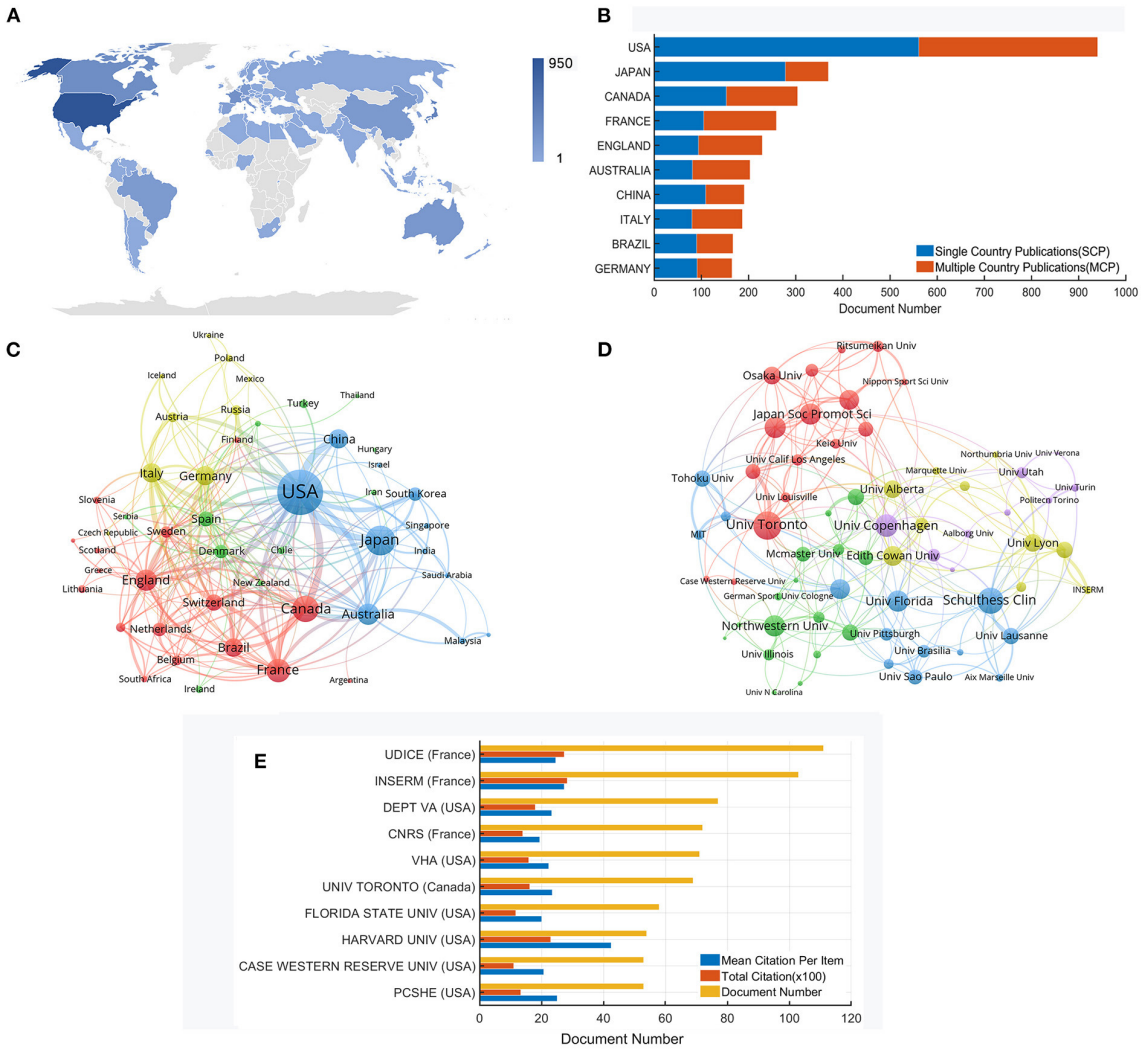
**(A)** Distribution of countries/regions that contributed to research (the shade of the color bar reflects the number of literature). **(B)** Publication collaboration of the top 10 productive countries. **(C)** Network map of global collaborative relationships. **(D)** Co-authorship network map of institutions. **(E)** Citation analysis of the top 10 productive affiliations.

All related papers were from 78 countries/regions. According to Table 2, the USA published the most papers (*n* = 940), accounting for 30.73%, followed by Japan (369 papers, 12.06%), Canada (304 papers, 9.94%), France (259 papers, 8.47%), and England (229 papers, 7.49%). In addition, the USA had significant advantages in total citations (*n* = 22,612), citations per paper (*n* = 24.06) and centrality (0.4). Figure 3A shows the distribution of countries/regions that contributed to the research, and Figure 3B shows the publication collaborations in different countries/regions. Global cooperation is shown in Figure 3C. There is strong cooperation among various countries. For instance, the USA frequently connects with Canada, France, England, Australia, and Italy.

**Table 2:** The top 10 productive countries/regions from 2011 to 2021.

**Rank**	**Country**	**Count (%)**	**Total citation**	**Citation per paper**	**Centrality**
1	USA	940 (30.73%)	22,612	24.06	0.40
2	Japan	369 (12.06%)	5,663	15.35	0.06
3	Canada	304 (9.94%)	6,165	20.28	0.07
4	France	259 (8.47%)	5,407	20.88	0.24
5	England	229 (7.49%)	4,696	20.51	0.20
6	Australia	203 (6.64%)	4,202	20.70	0.20
7	China	191 (6.24%)	4,077	21.35	0.02
8	Italy	187 (6.11%)	4,157	22.23	0.19
9	Brazil	167 (5.45%)	2,201	13.18	0.06
10	Germany	165 (5.39%)	4,301	26.07	0.08

A total of more than 2,789 institutions worldwide got involved. Figure 3D shows the collaborations among them. The University of Toronto, Schulthess Clin, University of Florida, University of Copenhagen, and Northwestern University are in larger circles on the network map, implying the importance of these institutions in the whole cooperation relationship. In the new version of the WoS database, the affiliation indicates the merged institution. The details of the top 10 prolific affiliations are shown in Figure 3E. Udice French Research Universities is ranked first, with 111 papers and 2,733 citations, followed by Institut National De La Sante Et De La Recherche Medicale (103 publications, 2,823 citations) and US Department of Veterans Affairs (77 publications, 1,796 citations). Harvard University reveals the highest citations per paper (*n* = 42.57). Furthermore, the top 10 affiliations locate in North America and Europe.

### Active authors and co-cited authors

**Figure 4:**
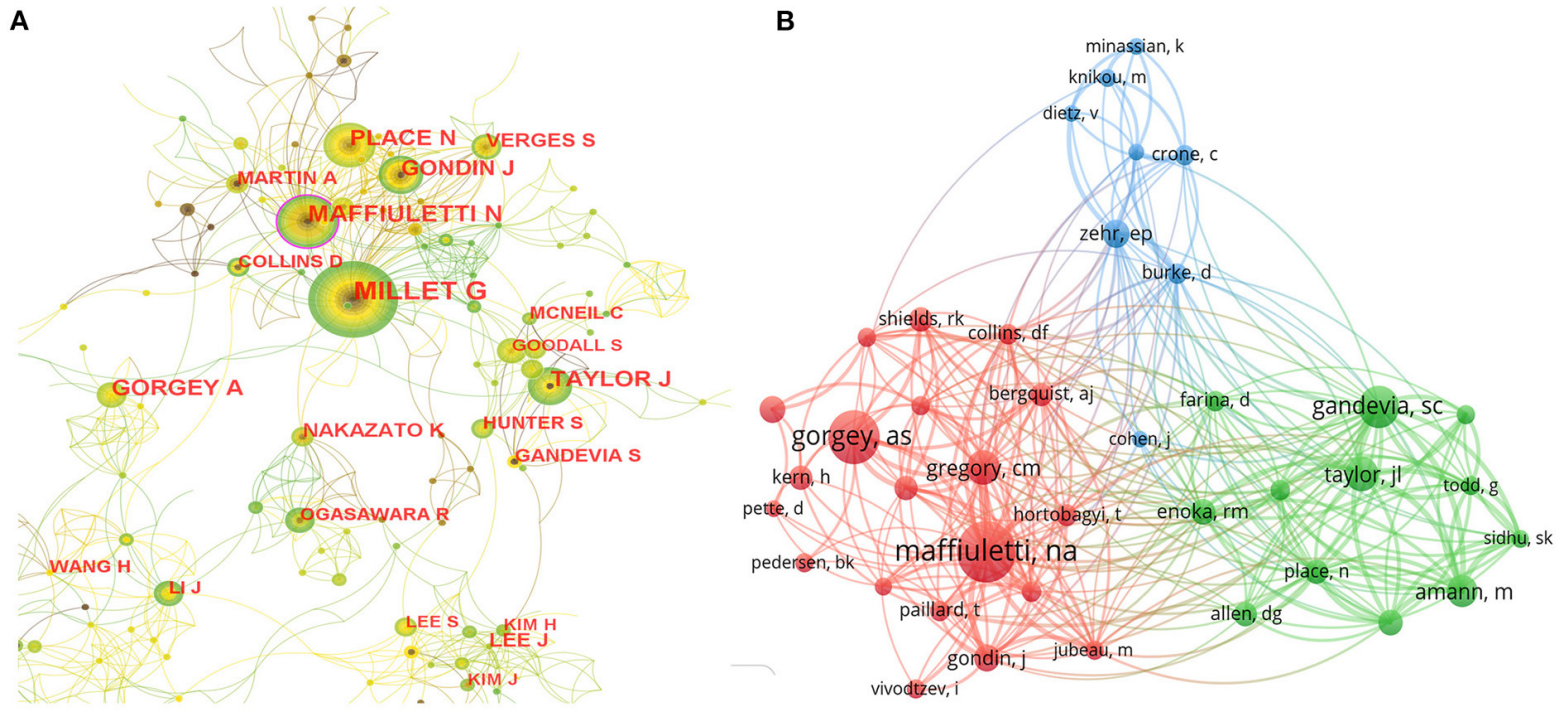
Collaboration network map of **(A)** authors and **(B)** co-cited authors.

A total of 11,377 authors participated in the studies. Table 3 lists the top 10 authors with the largest publications and co-cited authors with the highest citations. These authors are mainly from France or the USA. Millet GY is the most prolific author (*n* = 50), followed by Maffiuletti NA (*n* = 37) and Place *N* (*n* = 29). The collaboration network between authors is shown in Figure 4A. Authors from neighboring countries have close academic collaboration, but the connections between different continents are still weak. The co-cited author refers to the author who is also cited by other papers and constitutes a co-citation relationship. The degree of citation is a crucial index to measure the author's contribution. Figure 4B shows a network map of co-cited authors. Maffiuletti NA is the most frequently co-cited author with 489 citations.

**Table 3:** The top 10 prolific and co-cited authors from 2011 to 2021.

**Rank**	**Author**	**Counts**	**Citations**	**Country**	**Co-cited author**	**Citations**	**Country**
1	Millet GY	50	1,222	France	Maffiuletti NA	489	Switzerland
2	Maffiuletti NA	37	855	Switzerland	Gorgey AS	426	USA
3	Place *N*	29	554	Switzerland	Gandevia SC	319	Australia
4	Gorgey AS	29	554	USA	Gregory CM	272	USA
5	Gondin J	22	432	France	Taylor JL	272	Australia
6	Nakazato K	20	384	Japan	Amann M	245	USA
7	Verges S	19	613	France	Zehr EP	230	Canada
8	Taylor JL	19	485	Australia	Gondin J	201	France
9	Dixon WE	19	383	USA	Enoka RM	194	USA
10	Martin A	17	292	France	Kern H	190	Austria

### Distribution of journals and co-cited journals

All papers were published in 801 journals. Table 4 lists the most influential journals; half of them are from the USA. The number of publications in the top 10 journals varies from 39 to 104 (average 70), accounting for 22.69% of the total. Eight journals have impact factors above 3.000, publishing 18.96% of the whole papers. *PLOS ONE* published the most papers (*n* = 104), accounting for 3.40%, with 3,248 citations. *Medicine and Science in Sports and Exercise* had the highest impact factor (5.411) and average citations per paper (33.40).

**Table 4:** The top 10 journals and co-cited journals.

**Rank**	**Journal**	**Count**	**Citation/average citation**	**IF (2020)**	**JCR**	**Co-cited journal**	**Citation**	**IF (2020)**	**JCR**
1	Plos One (USA)	104 (3.40%)	3,248 (31.23)	3.24	Q2	Journal of Applied Physiology (USA)	7,192	3.532	Q2
2	European Journal of Applied Physiology (Germany)	96 (3.14%)	2,038 (21.23)	3.078	Q2	Journal of Physiology-London (England)	6,397	5.182	Q1
3	Journal of Applied Physiology (USA)	94 (3.07%)	1,831 (19.48)	3.532	Q2	Muscle Nerve (USA)	3,748	3.217	Q3
4	Muscle Nerve (USA)	84 (2.75%)	1,085 (12.92)	3.217	Q3	Journal of Neurophysiology (USA)	3,170	2.714	Q3
5	Journal of Neurophysiology (USA)	67 (2.19%)	1,157 (17.27)	2.714	Q3	European Journal of Applied Physiology (Germany)	2,912	3.078	Q2
6	Frontiers in Physiology (Switzerland)	67 (2.19%)	684 (10.21)	4.566	Q1	Medicine and Science in Sports and Exercise (USA)	2,629	5.411	Q1
7	Medicine and Science in Sports and Exercise (USA)	55 (1.80%)	1,837 (33.40)	5.411	Q1	Archives of Physical Medicine and Rehabilitation (USA)	2,609	3.966	Q1
8	Journal of Electromyography and Kinesiology (England)	47 (1.54%)	532 (11.32)	2.368	Q3	Experimental Brain Research (USA)	2,318	1.972	Q4
9	Journal of Physiology-London (England)	41 (1.34%)	999 (24.37)	5.182	Q1	Plos One (USA)	1,777	3.240	Q2
10	Scientific Reports (England)	39 (1.28%)	778 (19.95)	4.380	Q1	Physical Therapy (USA)	1,621	3.140	Q1

Table 4 also presents the top 10 co-cited journals. Two have more than 5,000 citations, and eight have impact factors above 3.000. The *Journal of Applied Physiology* from the USA is at the top, with 7,192 citations. The *Journal of Physiology-London* from England ranks second, with 6,397 citations.

Based on the 2020 Journal citation reports (JCR), more than half of the top 10 prolific journals and co-cited journals are in the Q2 and above region.

### Cited references and co-cited references

Table 5 summarizes the top 10 cited references. *What Is the Evidence for Physical Therapy Poststroke? A Systematic Review and Meta-Analysis*, published by Veerbeek et al. (38) in 2014, is the most cited paper, a total of 572 times. Another five references, published by Maltais et al. (39), Wagner et al. (40), Guo et al. (41), Zhang et al. (42), and Ajiboye et al. (43), have been cited more than 300 times. The remaining four highly cited papers are listed in the reference list (20, 44–46).

**Table 5:** The top 10 most cited references.

**Rank**	**Cited reference**	**Year**	**Citation**	**Journal**	**First author**
1	What Is the Evidence for Physical Therapy Poststroke? A Systematic Review and Meta-Analysis	2014	572	PLoS ONE	Veerbeek JM
2	An Official American Thoracic Society/European Respiratory Society Statement: Update on Limb Muscle Dysfunction in Chronic Obstructive Pulmonary Disease Executive Summary	2014	402	American Journal of Respiratory and Critical Care Medicine	Maltais F
3	Targeted neurotechnology restores walking in humans with spinal cord injury	2018	363	Nature	Wagner FB
4	Conducting Polymers for Tissue Engineering	2018	351	Biomacromolecules	Guo BL
5	A Human iPSC Model of Hutchinson Gilford Progeria Reveals Vascular Smooth Muscle and Mesenchymal Stem Cell Defects	2011	343	Cell stem cell	Zhang JQ
6	Restoration of reaching and grasping movements through brain-controlled muscle stimulation in a person with tetraplegia: a proof-of-concept demonstration	2017	337	Lancet	Ajiboye, AB
7	Restoration of grasp following paralysis through brain-controlled stimulation of muscles	2012	296	Nature	Ethier C
8	Interwoven Aligned Conductive Nanofiber Yarn/Hydrogel Composite Scaffolds for Engineered 3D Cardiac Anisotropy	2017	286	ACS nano	Wu YB
9	Physical Therapy for the Critically III in the ICU: A Systematic Review and Meta-Analysis	2013	271	Critical care medicine	Kayambu G
10	Biomaterials based strategies for skeletal muscle tissue engineering: Existing technologies and future trends	2015	254	Biomaterials	Qazi TH

**Figure 5:**
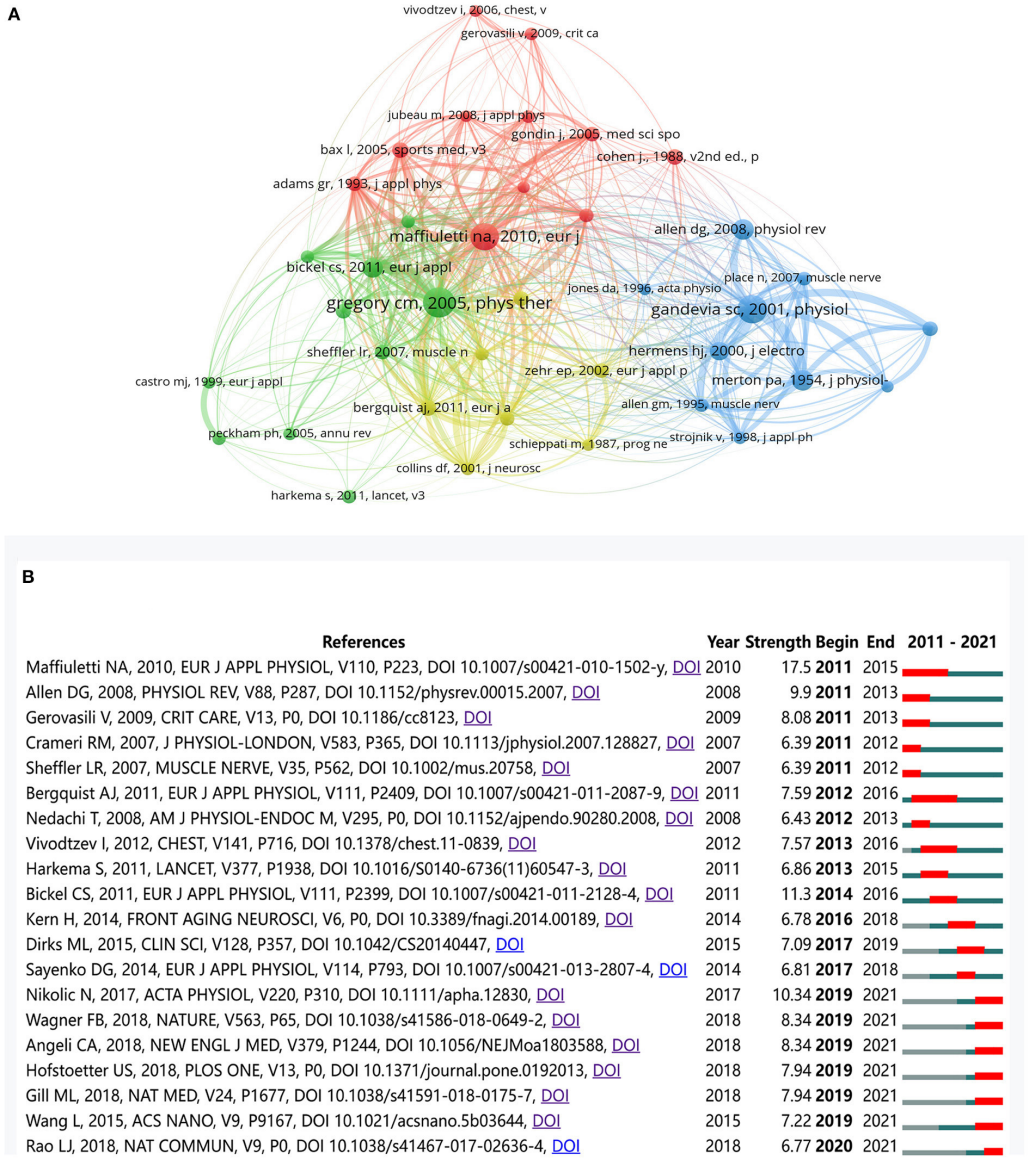
**(A)** The co-citation network map of references. **(B)** The top 20 references with the strongest citation bursts. The red segment denoted the burst duration of a reference.

A total of 94,666 co-cited references were identified. Figure 5A shows the network map of co-cited references. *Recruitment patterns in human skeletal muscle during electrical stimulation*, published by Gregory et al. (47). in 2005, is the most influential paper, indicating a solid citation relationship to the rest of the literature. The remaining references appearing in Figure 5A are listed in Supplement 1.

Furthermore, Figure 5B shows the top 20 references with the strongest citation bursts. These references contribute to the theoretical basis for research frontiers and reflect the evolution process in a particular field (48). “Maffiuletti NA (49), 2010, EUR J APPL PHYSIOL” has the strongest citation bursts (strength 17.50). A total of seven references have the most recent burst (2019–2021), and “Nikolic N (50), 2017, ACTA PHYSIO” is the strongest (strength 10.34) among them. The remaining references appearing in Figure 5B are listed in Supplement 2.

### Co-occurrence keywords and burst keywords

A total of 5,988 author keywords were extracted from 3,059 documents. The top 50 co-occurring keywords were identified in VOSviewer software. In order to accurately reveal the evolution of keywords, the subject terms of skeletal muscle and electrical stimulation, having the most frequent occurrence, without doubt, were excluded from the network analysis.

**Figure 6:**
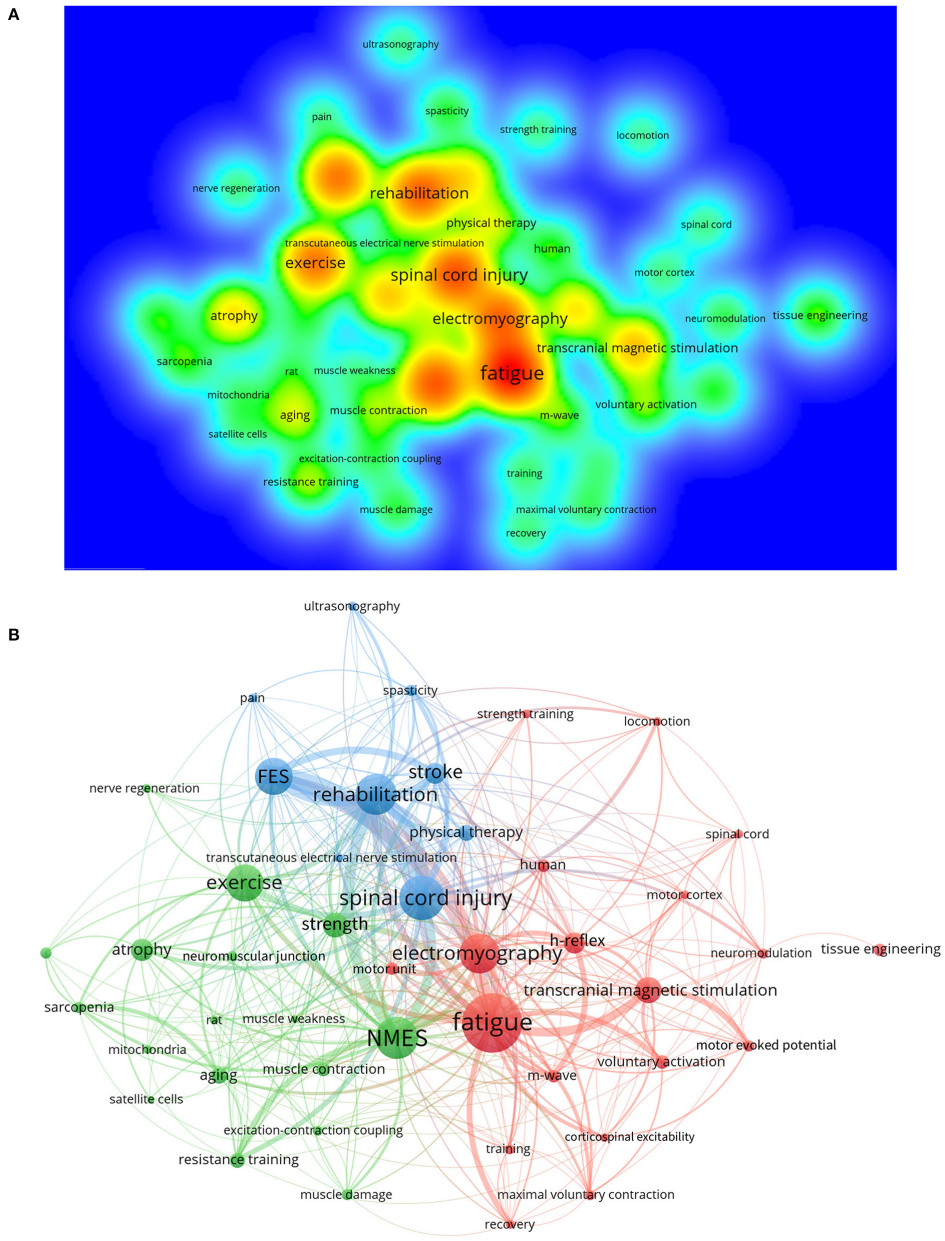
Visualization of keywords. **(A)** Density map of keywords co-occurrence. **(B)** Cluster network map. Nodes of the same color form a cluster.

Figure 6A shows the density visualization of the keywords and the hotspot intensity. The keywords with the highest density (located in warm red areas) are fatigue, spinal cord injury, NMES, rehabilitation, electromyography, exercise, FES, transcranial magnetic stimulation, strength, and atrophy.

In addition, Figure 6B shows the visualization of the co-occurrence network. There are 3 clusters to which the keywords belong. Cluster 1 (red) focuses on the treatment of muscle fatigue, including electrical stimulation, transcranial magnetic stimulation, or strength training. Electromyography, another central node, has connections with h-reflex, voluntary activation, m-wave, and motor unit, indicating that the monitoring and quantification of peripheral nerves, neurons, and muscle function are the topics of many studies. Furthermore, cluster 2 (green) concerns the application scenarios of NMES and exercise (resistance training), such as muscle atrophy, the aging process, sarcopenia, muscle damage, and weakness. These interventions may work at the molecular level, relating to the neuromuscular junction, excitation-contraction coupling, mitochondria, and satellite cells, and improve muscular strength and contraction. Finally, cluster 3 (blue) highlights the rehabilitation of clinical diseases, for instance, spinal cord injury and stroke, which result in severe muscle dysfunction. FES and transcutaneous electrical nerve stimulation, two types of physical therapies, are mentioned mainly in cluster 3.

**Figure 7:**
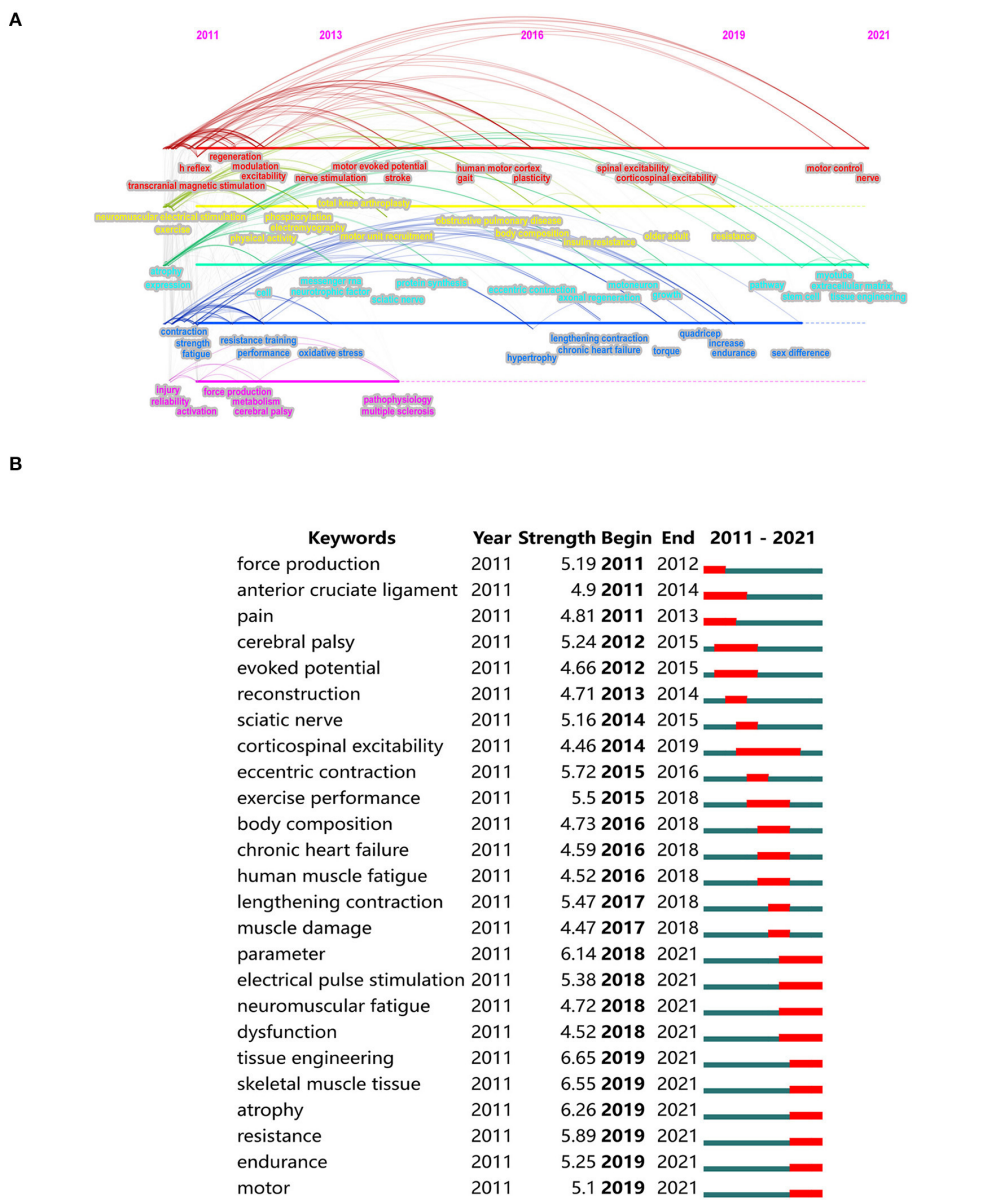
**(A)** Timeline viewer of keywords evolution. **(B)** The top 25 keywords with the strongest citation bursts.

Visible analysis of the phased hot topics is based on the timeline viewer (as shown in Figure 7A) from CiteSpace software. In the first five years (2011–2016), the studies focused on exploring the treatment of muscle function decline under pathological, fatigue, or injury conditions and its mechanism. The main keywords were: neuromuscular electrical stimulation, exercise, atrophy, stroke, obstructive pulmonary disease, motor cortex, plasticity, regeneration, neurotrophic factor, oxidative stress, and so on. The studies during 2016–2021 developed toward more specific aspects, with the keywords such as spinal excitability, motor control, older adult, sex difference, pathway, tissue engineering, endurance, contraction, and so on.

Keyword bursts also can be used to identify hot topics, frontiers, and trends in a specific research field. As shown in Figure 7B, the keywords with the most recent bursts are tissue engineering, skeletal muscle tissue, atrophy, parameter, resistance, and electrical pulse stimulation. The red lines in the diagram reflect the strongest citation bursts period. Corticospinal excitability is the keyword with the longest burst period, lasting five years (2014–2019). Table 6 lists the top five funding agencies and the most co-occurring keywords in the funded studies to further identify the funding and research topics.

**Table 6:** The top 5 funding agencies and co-occurrence keywords from publications.

**Funding agency**	**Count (%)**	**Country/region**	**Top co-occurrence keywords (frequency)**
United states department of health human services	427 (13.96%)	USA	Spinal cord injury (45), exercise (26), fatigue (26), functional electrical stimulation (26), electromyography (16), rehabilitation (16), aging (15), neuromuscular electrical stimulation (15).
National institutes of health	423 (13.83%)	USA	Spinal cord injury (44), functional electrical stimulation (26), exercise (25), fatigue (24), electromyography (16), aging (15), rehabilitation (15), neuromuscular electrical stimulation (15).
Ministry of education culture sports science and technology japan	237 (7.75%)	Japan	Exercise (16), atrophy (12), rehabilitation (11), h-reflex (8), muscle contraction (8), myokine (7), resistance training (7), spinal reflex (7).
Japan society for the promotion of science	192 (6.28%)	Japan	Exercise (13), rehabilitation (11), h-reflex (8), atrophy (7), muscle contraction (7), myokine (7), resistance training (7), spinal reflex (7).
European commission	143 (4.68%)	Europe	Exercise (12), electromyography (8), fatigue (8), neuromuscular electrical stimulation (7), atrophy (6), h-reflex (5), spinal cord injury (5), strength (5).

## Discussion

### Characteristics of the papers

This study provides a bibliometric analysis on electrical stimulation of skeletal muscle research over the past 10 years. The number of publications increased generally, but statistically, it remained at 230–335. This phenomenon indicates that it may have been a relatively mature research direction. Moreover, original research articles account for 86.35% of the total, followed by reviews (11.83%), implying a lot of new contributions to knowledge and summaries of the existing research. Neurosciences, Physiology, Sport Sciences, and Rehabilitation are the most prominent research area (as shown in Figure 2B). With the help of a dual-map overlay (as shown in Figure 2C), we find that molecular biology, genetics, sport, and rehabilitation are the primary research basis, and clinical medicine, neurology, and sports are significant application fields. Therefore, we speculate that these studies aim to understand better the response and adaptation of skeletal muscle to electrical stimulation in clinical pathological or neurological diseases and then explain the molecular mechanisms at the neuroscientific and physiological levels. Skeletal muscle is the supportive organ for daily exercise and physical activity, then maintaining the quality of human life. The effects of electrical stimulation on skeletal muscle function recovery and promotion are also worth noting.

### Geographical distribution of author and research group

A total of 11,377 authors contributed to the electrical stimulation of skeletal muscle research. However, according to statistics, only six authors published more than 20 papers, accounting for 0.08% of the total (references published by the top six productive authors are listed in Supplement 3); 66 authors published more than 10 papers, accounting for 0.58%. Most authors (*n* = 8,722, 76.78%) published only one paper. Although many researchers are engaged in the relevant work, only a small number of them have concentrated on this area.

Four of the top 10 prolific authors are from France, and two are from Switzerland. They are located in a densely connected local area within the cooperative network (as shown in Figure 4A), implying solid cooperative relationships. Millet GY is the leading author in the number of publications (*n* = 50) and citations (1,222 times). Four of the top 10 co-cited authors are from the USA. Analyzing the findings of the 10 authors, *Effects of Resistance Training on Adiposity and Metabolism after Spinal Cord Injury*, published by Gorgey in 2012 (51); *Motor unit recruitment during neuromuscular electrical stimulation: a critical appraisal*, published by Gregory in 2011 (52); *Group III/IV muscle afferents limit the intramuscular metabolic perturbation during whole body exercise in humans*, published by Amann in 2016 (53); and *Neural control of lengthening contractions*, published by Enoka in 2016 (54) are cited the most, more than 500 times. French and American scholars actively in this field and achieved high-quality research results.

According to Table 2, it is evident that the USA has advantages in the number of publications, citations, and centrality. Centrality is a measure of the significance of nodes in the network graph. Furthermore, the USA has extensive exchanges and cooperation with 50 countries, ranking first, followed by Australia (40 countries) and France (38 countries). All the evidence points out the dominance of the USA in global research. This is probably based on a large number of institutions and lots of research funding.

Only two of the top 10 productive countries are developing countries, China and Brazil. As far as the top 10 affiliations are concerned, six are from the USA, followed by France (3 affiliations). None of them locates in developing countries. Overall, the global influence of developing countries is limited in this field. As seen in Figure 3D, the location relationship among institutions is relatively scattered, indicating that worldwide academic cooperation is not strong enough. In order to promote mutual development, it is necessary to reinforce global communication and collaboration.

### Analysis of literature sources

A total of 3,059 papers were published in 801 journals. The top 10 prolific journals account for 22.69% of all publications. Most published articles distribute in other journals, indicating that many journals have been paying attention to the progress in this field.

Analysis of literature sources helps researchers find the core journals. As shown in Table 4, *Plos One* published the most papers, indicating its high popularity among scholars. However, the average citation of *Medicine and Science in Sports and Exercise* is the highest, reflecting the relatively good quality of papers published in this journal. In addition, eight journals are in the top 10 for productivity and co-citation. These journals may be good choices for researchers to present their work. Statistically, the most co-cited journal is the *Journal of Applied Physiology*, proving it has a good academic reputation in the industry.

The majority of the top 10 cited papers are original research, in general, involving the treatment of electrical stimulation on skeletal muscle dysfunction, design strategy, and application of conductive biomaterials in muscle tissue engineering. These studies reflect the concentrations of the researchers and provide empirical evidence for further research. Specifically, the most cited article is *What Is the Evidence for Physical Therapy Poststroke? A Systematic Review and Meta-Analysis*, providing an update of the evidence for stroke rehabilitation interventions in the domain of physical therapy, written by Veerbeek et al. (38). Following articles are related to the therapeutic effect of neuromuscular electrical stimulation written by Maltais et al. (39), and the re-establishment of adaptive control of paralyzed muscles using the invasive electrical stimulation after spinal cord injury, written by Wagner et al. (40), respectively.

### Analysis of the hot topics and trends

Based on co-word analysis combined with network analysis and keyword citation burst detection, hot topics and trends in this research field can be identified. Fatigue, NMES, exercise, rehabilitation, FES, and spinal cord injury are hot topics, and tissue engineering is the most citation-burst keyword recently, indicating the likely future trends.

### Effect of electrical stimulation on muscle fatigue

The performance and recovery of human muscle fatigue have recently been an important research topic (Figure 6B, cluster 1). Muscle fatigue typically presents with temporary strength loss (55, 56), such as a reduction of maximal voluntary contraction (MVC) (57). It will negatively affect the individual's motor ability and physical performance (58, 59), especially in competitive sports. There are both central and peripheral causes for the alterations of neuromuscular functional status. Recent research suggests that intramuscular inorganic phosphate is a primary cause of peripheral fatigue, skeletal muscle acidosis, probably acting on muscle afferents, as a contributor to central fatigue during exercise (60).

When it comes to restoration dynamics of skeletal muscle function, recovery strategies are highly required to alleviate fatigue, regain performance, and then reduce the risk of injury. The conventional idea is that these strategies should target the major causes of fatigue (61). For instance, hydration, diet, and sleep help replenish substrate stores and optimize muscle-damage repair. These interventions are effective in counteracting fatigue mechanisms. However, there is no consensus about the ability of electrical stimulation for a quick return to the initial level of muscle performance.

Babault et al. (62) summarized the effect of electrical stimulation on the ability to performance maintaining after exercise. They found that electrical stimulation did not work significantly in 11 of 12 studies. According to electromyography signals, a recent meta-analysis (63) suggests that electrical stimulation effectively reduces muscle fatigue during exercise but not a statistically significant effect. Even so, researchers do not deny the practical benefits of electrical stimulation (64–66). For instance, electrical stimulation helps reduce muscle soreness (67, 68) and enhances the clearance of creatine kinase and blood lactate (69, 70). In conclusion, electrical stimulation, as a common method for recovery purposes, the evidence concerning its therapeutic effects is limited. Additional studies are needed to establish efficient recovery protocols, particularly regarding the chronic effects, antiinflammatory or pro-inflammatory response, and combinations of recovery strategies.

NMES is commonly used to artificially control voluntary contractions of skeletal muscle (71, 72) and evaluate muscle performance or neuromuscular activation levels. The unique mechanism of NMES, but also a drawback, is the reversal of the regular voluntary recruitment pattern, which means the large and fatigable motor units are recruited earlier than smaller motor units (52). This phenomenon may limit practical applications because NMES tends to cause muscle fatigue (73). Michael et al. (74) demonstrated a new control modality for orderly recruitment to enhance performance and reduce fatigue. In order to achieve the maximum efficiency of NMES when applied to research, rehabilitation, and exercise, more scientific evidence is required, including the mechanism of orderly recruitment of motor units, parameters, and new technologies for NMES implementation (75). It will be a long-term goal in physiology, medicine, and engineering.

### FES for rehabilitation of paralytic skeletal muscles

There is a cross-linked network between the representative keywords of spinal cord injury, rehabilitation, FES, and stroke (Figure 6B, cluster 3). It indicates the formation of a specific research field and a research hotspot. Spinal cord injury (SCI) and stroke, although having different pathogenesis, are both critical factors causing paralysis. SCI can be traumatic (e.g., traffic accident) or non-traumatic (e.g., tumor). The connection between the central nervous system and the rest of the body breaks when the spinal cord is damaged, inducing a loss of sensory or motor ability. A lesion of the lumbar or thoracic levels leads to paraplegia, and the cervical level results in tetraplegia, which all seriously influence voluntary movement. Stroke is a partial death of brain tissue due to an interruption of the blood supply. Hemiplegia, impaired movement on one side of the body, is a common consequence of stroke. This condition can range from a slight decrease to severe damage or complete loss of motor capacity.

In recent years, the rehabilitation of voluntary movement has been enriched with the constant accumulation of neurophysiological evidence about the mechanism of motor function recovery (76). FES is an effective intervention that can assist functional and purposeful movements after SCI or stroke (77). FES therapy may promote adaptive plasticity, activate spare fibers, and stimulate central generators while bypassing damaged pathways. Since stimulus parameters influence the sequent movement, the safety, and the comfort of a person, confirming the parameters before using FES is essential. Electrode placement and stimulation intensity are two main aspects (9, 78). FES has been widely used in various rehabilitation programs. It improves walking and gripping and indirectly regulates cardiovascular health (79). Furthermore, FES also affects muscle fiber type transformation, metabolic gene activation, and diabetes risk factors reduction. However, there is contrary evidence that two weeks of FES-aided cycling would not contribute to immediate improvements in leg swelling or spasticity (80). The potential causes for these differences remain unclear.

With the aging of the population worldwide, the prevalence of FES is likely to increase over the coming years. Some research focuses on exploring new technology and strategy to deliver stimulation. Integrating FES and brain-computer interfaces (BCI) has been a hot global topic in neurorehabilitation (81). Furthermore, with the benefits of better mechanical compliance and lower skin irritation (82), the textile-based electrode for FES attracts the attention of researchers. In conclusion, more evidence is required to support effective FES protocols.

### Development of tissue engineering in skeletal muscle physiology

As shown in Figure 7B, tissue engineering is the keyword with the most citation burst, followed by skeletal muscle tissue and atrophy. Therefore, we briefly described the recent advances in tissue engineering in skeletal muscle physiology.

Based on the development of induced pluripotent stem cell (iPS) technology (83), tissue engineering enables the production of *in-vitro* tissue models (84). The artificial tissue model of skeletal muscle provides a platform to explore muscle physiology and the mechanism of muscle diseases (85). Muscle atrophy and contractile deficit are common complications of chronic inflammatory diseases. Exercise is a protective intervention, but its mechanism remains unclear. Chen et al. (86) verified that exercise-mimetic electrical stimulation attenuated interferon-γ (IFN-γ)–induced atrophy and weakness of the *in-vitro* skeletal muscle model. Specifically, the JAK (Janus kinase)/ STAT1 (signal transducer and activator of transcription 1) signaling pathway amplified by IFN-γ was down-regulated. Then the myobundle secretome altered, causing myofiber hypertrophy. Takahashi et al. (87) found that the periodic exercise induced by continuous electrical pulse stimulation strengthed the contractility of the engineered myofibers, and improved the level of interleukin-6 (IL-6) and vascular endothelial growth factor (VEGF). This type of engineered tissue can be used to better understand the relationship between mechanical stress and myogenesis. In addition, a number of studies focused on the optimization of *in-vitro* human skeletal muscle model (88, 89). These *in-vitro* models are expected to mimic real physiological properties, such as the alignment of muscle fibers, the basement structure of the extracellular matrix, and the contraction patterns. It is worth mentioning that electrical stimulation plays an important role both in inducing artificial movement of cells and forming mature neuromuscular junctions.

In summary, the tissue-engineered model of human skeletal muscle has become a powerful tool for studying myogenesis, metabolism, and the mechanisms of motoneurons and neuromuscular junction diseases. We believe that research on skeletal muscle tissue engineering will increase in the coming years.

## Conclusion

Over the past decade, the effects of electrical stimulation on the physiological function of skeletal muscle and neuromuscular diseases have attracted the attention of the academic community. Bibliometrics analysis helps scholars understand academic cooperation, research trends, and hot topics from numerous literatures.

In this study, we summarized the countries, affiliations, authors, journals, and citation data that contributed to the research on the electrical stimulation of skeletal muscle. Research groups from the USA and France are important contributors to the development of this field. In order to achieve more high-quality research results, international cooperation is supposed to strengthen. Based on the bibliometric review, the main hot topics and possible future directions are identified, for instance, the keywords of muscle fatigue, NMES, spinal cord injury, tissue engineering, and atrophy. Further studies are needed to explore new technology and application strategy to promote the development of this field.

## Data availability statement

The original contributions presented in the study are included in the article/Supplementary material, further inquiries can be directed to the corresponding author/s.

## Author contributions

Conception and design of the research: YH and JL. Acquisition of data, analysis, interpretation of data, and drafting the manuscript: YH and YG. Revision of the manuscript for important intellectual content: YL and JL. All authors have accepted responsibility for the entire content of this manuscript and approved its submission.

## Funding

This study was financially supported by the National Natural Science Foundation of China (No. 11932013, No. 31971102).

## Conflict of interest

The authors declare that the research was conducted in the absence of any commercial or financial relationships that could be construed as a potential conflict of interest.

## Publisher's note

All claims expressed in this article are solely those of the authors and do not necessarily represent those of their affiliated organizations, or those of the publisher, the editors and the reviewers. Any product that may be evaluated in this article, or claim that may be made by its manufacturer, is not guaranteed or endorsed by the publisher.
