# Supplementary material for: Global trends and hot topics in electrical stimulation of skeletal muscle research over the past decade: A bibliometric analysis
Source: Front Neurol. 2022 Oct 5;13:991099. doi: 10.3389/fneur.2022.991099 (PMC9581161; doi:10.3389/fneur.2022.991099)
Supplement: Supplementary file 1 [file Data_Sheet_1.DOCX]

# Supplement 1 The list of references in Figure 5A.

**Cluster 1**: red network (1-13).

1. Adams G, Harris R, Woodard D, Dudley GJJoap. Mapping of electrical muscle stimulation using MRI. (1993) 74:532-7.doi:10.1152/jappl.1993.74.2.532

2. Bax L, Staes F, Verhagen AJSm. Does neuromuscular electrical stimulation strengthen the quadriceps femoris? A systematic review of randomised controlled trials. (2005) 35:191-212.doi:10.2165/00007256-200535030-00002

3. Fujita H, Nedachi T, Kanzaki MJEcr. Accelerated de novo sarcomere assembly by electric pulse stimulation in C2C12 myotubes. (2007) 313:1853-65.doi:10.1016/j.yexcr.2007.03.002

4. Gerovasili V, Stefanidis K, Vitzilaios K, Karatzanos E, Politis P, Koroneos A, et al. Electrical muscle stimulation preserves the muscle mass of critically ill patients: a randomized study. (2009) 13:R161.doi:10.1186/cc8123

5. Gondin J, Guette M, Ballay Y, Martin AJM, sports si, exercise. Electromyostimulation training effects on neural drive and muscle architecture. (2005) 37:1291-9.doi:10.1249/01.mss.0000175090.49048.41

6. Gondin J, Brocca L, Bellinzona E, D'Antona G, Maffiuletti N, Miotti D, et al. Neuromuscular electrical stimulation training induces atypical adaptations of the human skeletal muscle phenotype: a functional and proteomic analysis. (2011) 110:433-50.doi:10.1152/japplphysiol.00914.2010

7. Jubeau M, Sartorio A, Marinone P, Agosti F, Van Hoecke J, Nosaka K, et al. Comparison between voluntary and stimulated contractions of the quadriceps femoris for growth hormone response and muscle damage. (2008) 104:75-81.doi:10.1152/japplphysiol.00335.2007

8. Maffiuletti NJEjoap. Physiological and methodological considerations for the use of neuromuscular electrical stimulation. (2010) 110:223-34.doi:10.1007/s00421-010-1502-y

9. Nedachi T, Fujita H, Kanzaki MJAjopE, metabolism. Contractile C2C12 myotube model for studying exercise-inducible responses in skeletal muscle. (2008) 295:E1191-204.doi:10.1152/ajpendo.90280.2008

10. Vanderthommen M, Duteil S, Wary C, Raynaud J, Leroy-Willig A, Crielaard J, et al. A comparison of voluntary and electrically induced contractions by interleaved 1H- and 31P-NMRS in humans. (2003) 94:1012-24.doi:10.1152/japplphysiol.00887.2001

11. Vanderthommen M, Duchateau JJE, reviews ss. Electrical stimulation as a modality to improve performance of the neuromuscular system. (2007) 35:180-5.doi:10.1097/jes.0b013e318156e785

12. Vivodtzev I, Pépin J, Vottero G, Mayer V, Porsin B, Lévy P, et al. Improvement in quadriceps strength and dyspnea in daily tasks after 1 month of electrical stimulation in severely deconditioned and malnourished COPD. (2006) 129:1540-8.doi:10.1378/chest.129.6.1540

13. Cohen J, Malter H, Fehilly C, Wright G, Elsner C, Kort H, et al. Implantation of embryos after partial opening of oocyte zona pellucida to facilitate sperm penetration. Lancet (London, England). (1988) 2:162.doi:10.1016/s0140-6736(88)90710-6

**Cluster 2**: blue network (14-23).

14. Allen D, Lamb G, Westerblad HJPr. Skeletal muscle fatigue: cellular mechanisms. (2008) 88:287-332.doi:10.1152/physrev.00015.2007

15. Allen G, Gandevia S, McKenzie DJM, nerve. Reliability of measurements of muscle strength and voluntary activation using twitch interpolation. (1995) 18:593-600.doi:10.1002/mus.880180605

16. Gandevia S, Allen G, Butler J, Taylor JJTJop. Supraspinal factors in human muscle fatigue: evidence for suboptimal output from the motor cortex. (1996) 529-36.doi:10.1113/jphysiol.1996.sp021164

17. Gandevia SJPr. Spinal and supraspinal factors in human muscle fatigue. (2001) 81:1725-89.doi:10.1152/physrev.2001.81.4.1725

18. Jones DJApS. High-and low-frequency fatigue revisited. (1996) 156:265-70.doi:10.1046/j.1365-201X.1996.192000.x

19. Hermens HJ, Freriks B, Disselhorst-Klug C, Rau G. Development of recommendations for SEMG sensors and sensor placement procedures. Journal of electromyography and kinesiology : official journal of the International Society of Electrophysiological Kinesiology. (2000) 10:361-74.doi:10.1016/s1050-6411(00)00027-4

20. MERTON PJTJop. Voluntary strength and fatigue. (1954) 123:553-64.doi:10.1113/jphysiol.1954.sp005070

21. Place N, Maffiuletti N, Martin A, Lepers RJM, nerve. Assessment of the reliability of central and peripheral fatigue after sustained maximal voluntary contraction of the quadriceps muscle. (2007) 35:486-95.doi:10.1002/mus.20714

22. Strojnik V, Komi PJJoap. Neuromuscular fatigue after maximal stretch-shortening cycle exercise. (1998) 84:344-50.doi:10.1152/jappl.1998.84.1.344

23. Todd G, Taylor JL, Gandevia SC. Measurement of voluntary activation of fresh and fatigued human muscles using transcranial magnetic stimulation. The Journal of physiology. (2003) 551:661-71.doi:10.1113/jphysiol.2003.044099

**Cluster 3**: green network (24-33).

24. Bickel C, Gregory C, Dean JJEjoap. Motor unit recruitment during neuromuscular electrical stimulation: a critical appraisal. (2011) 111:2399-407.doi:10.1007/s00421-011-2128-4

25. Castro M, Apple D, Hillegass E, Dudley GJEjoap, physiology o. Influence of complete spinal cord injury on skeletal muscle cross-sectional area within the first 6 months of injury. (1999) 80:373-8.doi:10.1007/s004210050606

26. Doucet BM, Lam A, Griffin L. Neuromuscular electrical stimulation for skeletal muscle function. The Yale journal of biology and medicine. (2012) 85:201-15

27. Gorgey A, Dudley GJSc. Skeletal muscle atrophy and increased intramuscular fat after incomplete spinal cord injury. (2007) 45:304-9.doi:10.1038/sj.sc.3101968

28. Gorgey A, Black C, Elder C, Dudley GJTJoo, therapy sp. Effects of electrical stimulation parameters on fatigue in skeletal muscle. (2009) 39:684-92.doi:10.2519/jospt.2009.3045

29. Gregory CM, Bickel CS. Recruitment patterns in human skeletal muscle during electrical stimulation. Physical therapy. (2005) 85:358-64

30. Gregory C, Dixon W, Bickel CJM, nerve. Impact of varying pulse frequency and duration on muscle torque production and fatigue. (2007) 35:504-9.doi:10.1002/mus.20710

31. Harkema S, Gerasimenko Y, Hodes J, Burdick J, Angeli C, Chen Y, et al. Effect of epidural stimulation of the lumbosacral spinal cord on voluntary movement, standing, and assisted stepping after motor complete paraplegia: a case study. Lancet (London, England). (2011) 377:1938-47.doi:10.1016/s0140-6736(11)60547-3

32. Peckham PH, Knutson JS. Functional electrical stimulation for neuromuscular applications. Annual review of biomedical engineering. (2005) 7:327-60.doi:10.1146/annurev.bioeng.6.040803.140103

33. Sheffler LR, Chae J. Neuromuscular electrical stimulation in neurorehabilitation. Muscle & nerve. (2007) 35:562-90.doi:10.1002/mus.20758

**Cluster 4**: yellow network (34-40).

34. Bergquist AJ, Clair JM, Lagerquist O, Mang CS, Okuma Y, Collins DF. Neuromuscular electrical stimulation: implications of the electrically evoked sensory volley. European journal of applied physiology. (2011) 111:2409-26.doi:10.1007/s00421-011-2087-9

35. Collins DF, Burke D, Gandevia SC. Large involuntary forces consistent with plateau-like behavior of human motoneurons. The Journal of neuroscience : the official journal of the Society for Neuroscience. (2001) 21:4059-65.doi:10.1523/jneurosci.21-11-04059.2001

36. Collins DF, Burke D, Gandevia SC. Sustained contractions produced by plateau-like behaviour in human motoneurones. The Journal of physiology. (2002) 538:289-301.doi:10.1113/jphysiol.2001.012825

37. Collins DF. Central contributions to contractions evoked by tetanic neuromuscular electrical stimulation. Exercise and sport sciences reviews. (2007) 35:102-9.doi:10.1097/jes.0b013e3180a0321b

38. Henneman E, Somjen G, Carpenter DO. FUNCTIONAL SIGNIFICANCE OF CELL SIZE IN SPINAL MOTONEURONS. Journal of neurophysiology. (1965) 28:560-80.doi:10.1152/jn.1965.28.3.560

39. Schieppati M. The Hoffmann reflex: a means of assessing spinal reflex excitability and its descending control in man. Progress in neurobiology. (1987) 28:345-76.doi:10.1016/0301-0082(87)90007-4

40. Zehr EP. Considerations for use of the Hoffmann reflex in exercise studies. European journal of applied physiology. (2002) 86:455-68.doi:10.1007/s00421-002-0577-5

**Supplement 2** The list of references in Figure 5B (1-20).

1. Maffiuletti NJEjoap. Physiological and methodological considerations for the use of neuromuscular electrical stimulation. (2010) 110:223-34.doi:10.1007/s00421-010-1502-y

2. Allen D, Lamb G, Westerblad HJPr. Skeletal muscle fatigue: cellular mechanisms. (2008) 88:287-332.doi:10.1152/physrev.00015.2007

3. Gerovasili V, Stefanidis K, Vitzilaios K, Karatzanos E, Politis P, Koroneos A, et al. Electrical muscle stimulation preserves the muscle mass of critically ill patients: a randomized study. Critical Care. (2009) 13:R161.doi:10.1186/cc8123

4. Crameri RM, Aagaard P, Qvortrup K, Langberg H, Olesen J, Kjær M. Myofibre damage in human skeletal muscle: effects of electrical stimulation versus voluntary contraction. (2007) 583:365-80.doi:<https://doi.org/10.1113/jphysiol.2007.128827>

5. Sheffler LR, Chae J. Neuromuscular electrical stimulation in neurorehabilitation. Muscle & nerve. (2007) 35:562-90.doi:10.1002/mus.20758

6. Bergquist AJ, Clair JM, Lagerquist O, Mang CS, Okuma Y, Collins DF. Neuromuscular electrical stimulation: implications of the electrically evoked sensory volley. European journal of applied physiology. (2011) 111:2409-26.doi:10.1007/s00421-011-2087-9

7. Nedachi T, Fujita H, Kanzaki MJAjopE, metabolism. Contractile C2C12 myotube model for studying exercise-inducible responses in skeletal muscle. (2008) 295:E1191-204.doi:10.1152/ajpendo.90280.2008

8. Vivodtzev I, Debigaré R, Gagnon P, Mainguy V, Saey D, Dubé A, et al. Functional and Muscular Effects of Neuromuscular Electrical Stimulation in Patients With Severe COPD: A Randomized Clinical Trial. Chest. (2012) 141:716-25.doi:<https://doi.org/10.1378/chest.11-0839>

9. Harkema S, Gerasimenko Y, Hodes J, Burdick J, Angeli C, Chen Y, et al. Effect of epidural stimulation of the lumbosacral spinal cord on voluntary movement, standing, and assisted stepping after motor complete paraplegia: a case study. Lancet (London, England). (2011) 377:1938-47.doi:10.1016/s0140-6736(11)60547-3

10. Bickel CS, Gregory CM, Dean JC. Motor unit recruitment during neuromuscular electrical stimulation: a critical appraisal. European journal of applied physiology. (2011) 111:2399-407.doi:10.1007/s00421-011-2128-4

11. Kern H, Barberi L, Lofler S, Sbardella S, Burggraf S, Fruhmann H, et al. Electrical stimulation counteracts muscle decline in seniors. Front Aging Neurosci. (2014) 6:189.doi:10.3389/fnagi.2014.00189

12. Dirks Marlou L, Hansen D, Van Assche A, Dendale P, Van Loon Luc JC. Neuromuscular electrical stimulation prevents muscle wasting in critically ill comatose patients. Clinical Science. (2014) 128:357-65.doi:10.1042/CS20140447 %J Clinical Science

13. Sayenko DG, Nguyen R, Popovic MR, Masani K. Reducing muscle fatigue during transcutaneous neuromuscular electrical stimulation by spatially and sequentially distributing electrical stimulation sources. European journal of applied physiology. (2014) 114:793-804.doi:10.1007/s00421-013-2807-4

14. Nikolić N, Görgens SW, Thoresen GH, Aas V, Eckel J, Eckardt K. Electrical pulse stimulation of cultured skeletal muscle cells as a model for in vitro exercise - possibilities and limitations. Acta physiologica (Oxford, England). (2017) 220:310-31.doi:10.1111/apha.12830

15. Wagner FB, Mignardot JB, Le Goff-Mignardot CG, Demesmaeker R, Komi S, Capogrosso M, et al. Targeted neurotechnology restores walking in humans with spinal cord injury. Nature. (2018) 563:65-71.doi:10.1038/s41586-018-0649-2

16. Angeli CA, Boakye M, Morton RA, Vogt J, Benton K, Chen Y, et al. Recovery of Over-Ground Walking after Chronic Motor Complete Spinal Cord Injury. (2018) 379:1244-50.doi:10.1056/NEJMoa1803588

17. Hofstoetter US, Freundl B, Binder H, Minassian K. Common neural structures activated by epidural and transcutaneous lumbar spinal cord stimulation: Elicitation of posterior root-muscle reflexes. PLoS One. (2018) 13:e0192013.doi:10.1371/journal.pone.0192013

18. Gill ML, Grahn PJ, Calvert JS, Linde MB, Lavrov IA, Strommen JA, et al. Neuromodulation of lumbosacral spinal networks enables independent stepping after complete paraplegia. Nature medicine. (2018) 24:1677-82.doi:10.1038/s41591-018-0175-7

19. Wang L, Wu Y, Guo B, Ma PX. Nanofiber Yarn/Hydrogel Core–Shell Scaffolds Mimicking Native Skeletal Muscle Tissue for Guiding 3D Myoblast Alignment, Elongation, and Differentiation. ACS Nano. (2015) 9:9167-79.doi:10.1021/acsnano.5b03644

20. Rao L, Qian Y, Khodabukus A, Ribar T, Bursac N. Engineering human pluripotent stem cells into a functional skeletal muscle tissue. Nature Communications. (2018) 9:126.doi:10.1038/s41467-017-02636-4

# Supplement 3 The list of references published by the top six productive authors.

Millet GY (1-51);

Maffiuletti NA (11, 16, 38, 52-85);

Place N (38, 39, 59, 60, 67, 71-73, 75, 76, 80, 82-84, 86-100);

Gorgey AS (101-128);

Gondin J (16, 28, 59, 61, 71, 80, 82, 83, 91, 129-142);

Nakazato K (143-162).

1. Aboodarda SJ, Fan S, Coates K, Millet GY. The short-term recovery of corticomotor responses in elbow flexors. BMC NEUROSCIENCE. (2019) 20.doi:10.1186/s12868-019-0492-x

2. Aboodarda SJ, Greene RM, Philpott DT, Jaswal RS, Millet GY, Behm DG. The effect of rolling massage on the excitability of the corticospinal pathway. APPLIED PHYSIOLOGY NUTRITION AND METABOLISM. (2018) 43:317-23.doi:10.1139/apnm-2017-0408

3. Aboodarda SJ, Sambaher N, Millet GY, Behm DG. KNEE EXTENSORS NEUROMUSCULAR FATIGUE CHANGES THE CORTICOSPINAL PATHWAY EXCITABILITY IN BICEPS BRACHII MUSCLE. NEUROSCIENCE. (2017) 340:477-86.doi:10.1016/j.neuroscience.2016.10.065

4. Arnal PJ, Lapole T, Erblang M, Guillard M, Bourrilhon C, Leger D, et al. Sleep Extension before Sleep Loss: Effects on Performance and Neuromuscular Function. Medicine and science in sports and exercise. (2016) 48:1595-603.doi:10.1249/MSS.0000000000000925

5. Azevedo RD, Forot J, Iannetta D, MacInnis MJ, Millet GY, Murias JM. Slight power output manipulations around the maximal lactate steady state have a similar impact on fatigue in females and males. JOURNAL OF APPLIED PHYSIOLOGY. (2021) 130:1879-92.doi:10.1152/japplphysiol.00892.2020

6. Bachasson D, Guinot M, Wuyam B, Favre-Juvin A, Millet GY, Levy P, et al. Neuromuscular Fatigue and Exercise Capacity in Fibromyalgia Syndrome. ARTHRITIS CARE & RESEARCH. (2013) 65:432-40.doi:10.1002/acr.21845

7. Bachasson D, Millet GY, Decorte N, Wuyam B, Levy P, Verges S. Quadriceps function assessment using an incremental test and magnetic neurostimulation: A reliability study. JOURNAL OF ELECTROMYOGRAPHY AND KINESIOLOGY. (2013) 23:649-58.doi:10.1016/j.jelekin.2012.11.011

8. Bachasson D, Temesi J, Bankole C, Lagrange E, Boutte C, Millet GY, et al. Assessement of quadriceps strength, endurance and fatigue in FSHD and CMT: Benefits and limits of femoral nerve magnetic stimulation. CLINICAL NEUROPHYSIOLOGY. (2014) 125:396-405.doi:10.1016/j.clinph.2013.08.001

9. Besson T, Parent A, Brownstein C, Espeit L, Lapole T, Martin V, et al. Sex Differences in Neuromuscular Fatigue and Changes in Cost of Running after Mountain Trail Races of Various Distances. MEDICINE & SCIENCE IN SPORTS & EXERCISE. (2021) 53:2374-87.doi:10.1249/MSS.0000000000002719

10. Besson T, Rossi J, Mallouf TL, Marechal M, Doutreleau S, Verges S, et al. Fatigue and Recovery after Single-Stage versus Multistage Ultramarathon Running. MEDICINE & SCIENCE IN SPORTS & EXERCISE. (2020) 52:1691-8.doi:10.1249/MSS.0000000000002303

11. Blazevich AJ, Collins DF, Millet GY, Vaz MA, Maffiuletti NA. Enhancing Adaptations to Neuromuscular Electrical Stimulation Training Interventions. Exercise and sport sciences reviews. (2021) 49:244-52.doi:10.1249/JES.0000000000000264

12. Brownstein CG, Millet GY, Thomas K. Neuromuscular responses to fatiguing locomotor exercise. ACTA PHYSIOLOGICA. (2021) 231.doi:10.1111/apha.13533

13. Coates KD, Aboodarda SJ, Kruger RL, Martin T, Metz LM, Jarvis SE, et al. Multiple sclerosis-related fatigue: the role of impaired corticospinal responses and heightened exercise fatigability. Journal of neurophysiology. (2020) 124:1131-43.doi:10.1152/jn.00165.2020

14. Doyle-Baker D, Temesi J, Medysky ME, Holash RJ, Millet GY. An Innovative Ergometer to Measure Neuromuscular Fatigue Immediately after Cycling. Medicine and science in sports and exercise. (2018) 50:375-87.doi:10.1249/MSS.0000000000001427

15. Espeit L, Brownstein CG, Royer N, Besson T, Martin V, Millet GY, et al. Central fatigue aetiology in prolonged trail running races. EXPERIMENTAL PHYSIOLOGY. (2021) 106:663-72.doi:10.1113/EP089177

16. Espeit L, Rozand V, Millet GY, Gondin J, Maffiuletti NA, Lapole T. Influence of wide-pulse neuromuscular electrical stimulation frequency and superimposed tendon vibration on occurrence and magnitude of extra torque. JOURNAL OF APPLIED PHYSIOLOGY. (2021) 131:302-12.doi:10.1152/japplphysiol.00968.2020

17. Froyd C, Beltrami FG, Jensen J, Millet GY, Noakes TD. Potentiation and Electrical Stimulus Frequency During Self-Paced Exercise and Recovery. JOURNAL OF HUMAN KINETICS. (2014) 42:91-101.doi:10.2478/hukin-2014-0064

18. Froyd C, Beltrami FG, Millet GY, MacIntosh BR, Noakes TD. Greater Short-Time Recovery of Peripheral Fatigue After Short- Compared With Long-Duration Time Trial. FRONTIERS IN PHYSIOLOGY. (2020) 11.doi:10.3389/fphys.2020.00399

19. Froyd C, Beltrami FG, Millet GY, Noakes TD. Central Regulation and Neuromuscular Fatigue during Exercise of Different Durations. Medicine and science in sports and exercise. (2016) 48:1024-32.doi:10.1249/MSS.0000000000000867

20. Froyd C, Beltrami FG, Millet GY, Noakes TD. No Critical Peripheral Fatigue Threshold during Intermittent Isometric Time to Task Failure Test with the Knee Extensors. FRONTIERS IN PHYSIOLOGY. (2016) 7.doi:10.3389/fphys.2016.00627

21. Froyd C, Millet GY, Noakes TD. The development of peripheral fatigue and short-term recovery during self-paced high-intensity exercise. JOURNAL OF PHYSIOLOGY-LONDON. (2013) 591:1339-46.doi:10.1113/jphysiol.2012.245316

22. Giandolini M, Horvais N, Rossi J, Millet GY, Morin JB, Samozino P. Acute and delayed peripheral and central neuromuscular alterations induced by a short and intense downhill trail run. SCANDINAVIAN JOURNAL OF MEDICINE & SCIENCE IN SPORTS. (2016) 26:1321-33.doi:10.1111/sms.12583

23. Grenier JG, Millet GY, Peyrot N, Samozino P, Oullion R, Messonnier L, et al. Effects of Extreme-Duration Heavy Load Carriage on Neuromuscular Function and Locomotion: A Military-Based Study. PLOS ONE. (2012) 7.doi:10.1371/journal.pone.0043586

24. Gruet M, Temesi J, Rupp T, Levy P, Verges S, Millet GY. Dynamics of corticospinal changes during and after high-intensity quadriceps exercise. EXPERIMENTAL PHYSIOLOGY. (2014) 99:1053-64.doi:10.1113/expphysiol.2014.078840

25. Hureau TJ, Olivier N, Millet GY, Meste O, Blain GM. Exercise performance is regulated during repeated sprints to limit the development of peripheral fatigue beyond a critical threshold. EXPERIMENTAL PHYSIOLOGY. (2014) 99:951-63.doi:10.1113/expphysiol.2014.077974

26. Jubeau M, Rupp T, Perrey S, Temesi J, Wuyam B, Levy P, et al. Changes in Voluntary Activation Assessed by Transcranial Magnetic Stimulation during Prolonged Cycling Exercise. PLOS ONE. (2014) 9.doi:10.1371/journal.pone.0089157

27. Jubeau M, Rupp T, Temesi J, Perrey S, Wuyam B, Millet GY, et al. Neuromuscular Fatigue during Prolonged Exercise in Hypoxia. MEDICINE & SCIENCE IN SPORTS & EXERCISE. (2017) 49:430-9.doi:10.1249/MSS.0000000000001118

28. Kennouche D, Luneau E, Lapole T, Morel J, Millet GY, Gondin J. Bedside voluntary and evoked forces evaluation in intensive care unit patients: a narrative review. CRITICAL CARE. (2021) 25.doi:10.1186/s13054-021-03567-9

29. Kruger RL, Aboodarda SJ, Jaimes LM, Samozino P, Millet GY. Cycling performed on an innovative ergometer at different intensities-durations in men: neuromuscular fatigue and recovery kinetics. APPLIED PHYSIOLOGY NUTRITION AND METABOLISM. (2019) 44:1320-8.doi:10.1139/apnm-2018-0858

30. Lapole T, Temesi J, Arnal PJ, Gimenez P, Petitjean M, Millet GY. Modulation of soleus corticospinal excitability during Achilles tendon vibration. EXPERIMENTAL BRAIN RESEARCH. (2015) 233:2655-62.doi:10.1007/s00221-015-4336-3

31. Lavigne C, Lau H, Francis G, Culos-Reed SN, Millet GY, Twomey R. Neuromuscular function and fatigability in people diagnosed with head and neck cancer before versus after treatment. European journal of applied physiology. (2020) 120:1289-304.doi:10.1007/s00421-020-04362-0

32. Lavigne C, Twomey R, Lau H, Francis G, Culos-Reed SN, Millet GY. Feasibility of eccentric overloading and neuromuscular electrical stimulation to improve muscle strength and muscle mass after treatment for head and neck cancer. JOURNAL OF CANCER SURVIVORSHIP. (2020) 14:790-805.doi:10.1007/s11764-020-00893-9

33. Marillier M, Arnal PJ, Mallouf TL, Rupp T, Millet GY, Verges S. Effects of high-altitude exposure on supraspinal fatigue and corticospinal excitability and inhibition. European journal of applied physiology. (2017) 117:1747-61.doi:10.1007/s00421-017-3669-y

34. Millet GY. Can Neuromuscular Fatigue Explain Running Strategies and Performance in Ultra-Marathons? The Flush Model. SPORTS MEDICINE. (2011) 41:489-506.doi:10.2165/11588760-000000000-00000

35. Millet GY, Bachasson D, Temesi J, Wuyam B, Feasson L, Verges S, et al. Potential interests and limits of magnetic and electrical stimulation techniques to assess neuromuscular fatigue. NEUROMUSCULAR DISORDERS. (2012) 22:S181-S6.doi:10.1016/j.nmd.2012.10.007

36. Millet GY, Martin V, Martin A, Verges S. Electrical stimulation for testing neuromuscular function: from sport to pathology. European journal of applied physiology. (2011) 111:2489-500.doi:10.1007/s00421-011-1996-y

37. Mira J, Aboodarda SJ, Floreani M, Jaswal R, Moon SJ, Amery K, et al. Effects of endurance training on neuromuscular fatigue in healthy active men. Part I: Strength loss and muscle fatigue. European journal of applied physiology. (2018) 118:2281-93.doi:10.1007/s00421-018-3950-8

38. Neyroud D, Temesi J, Millet GY, Verges S, Maffiuletti NA, Kayser B, et al. Comparison of electrical nerve stimulation, electrical muscle stimulation and magnetic nerve stimulation to assess the neuromuscular function of the plantar flexor muscles. European journal of applied physiology. (2015) 115:1429-39.doi:10.1007/s00421-015-3124-x

39. Neyroud D, Vallotton A, Millet GY, Kayser B, Place N. The effect of muscle fatigue on stimulus intensity requirements for central and peripheral fatigue quantification. European journal of applied physiology. (2014) 114:205-15.doi:10.1007/s00421-013-2760-2

40. Peyrard A, Sawh P, Fan S, Temesi J, Millet GY. Anticipation of magnetic and electrical stimuli does not impair maximal voluntary force production. NEUROSCIENCE LETTERS. (2016) 628:128-31.doi:10.1016/j.neulet.2016.06.024

41. Place N, Millet GY. Quantification of Neuromuscular Fatigue: What Do We Do Wrong and Why? SPORTS MEDICINE. (2020) 50:439-47.doi:10.1007/s40279-019-01203-9

42. Rupp T, Jubeau M, Wuyam B, Perrey S, Levy P, Millet GY, et al. Time-dependent effect of acute hypoxia on corticospinal excitability in healthy humans. Journal of neurophysiology. (2012) 108:1270-7.doi:10.1152/jn.01162.2011

43. Rupp T, Mallouf TL, Perrey S, Wuyam B, Millet GY, Verges S. CO2 Clamping, Peripheral and Central Fatigue during Hypoxic Knee Extensions in Men. Medicine and science in sports and exercise. (2015) 47:2513-24.doi:10.1249/MSS.0000000000000724

44. Souron R, Baudry S, Millet GY, Lapole T. Vibration-induced depression in spinal loop excitability revisited. JOURNAL OF PHYSIOLOGY-LONDON. (2019) 597:5179-93.doi:10.1113/JP278469

45. Souron R, Besson T, McNeil CJ, Lapole T, Millet GY. An Acute Exposure to Muscle Vibration Decreases Knee Extensors Force Production and Modulates Associated Central Nervous System Excitability. FRONTIERS IN HUMAN NEUROSCIENCE. (2017) 11.doi:10.3389/fnhum.2017.00519

46. Souron R, Morel J, Gergele L, Infantino P, Brownstein CG, Lapole T, et al. Relationship between intensive care unit-acquired weakness, fatigability and fatigue: What role for the central nervous system? JOURNAL OF CRITICAL CARE. (2021) 62:101-10.doi:10.1016/j.jcrc.2020.11.019

47. Temesi J, Arnal PJ, Rupp T, Feasson L, Cartier R, Gergele L, et al. Are Females More Resistant to Extreme Neuromuscular Fatigue? Medicine and science in sports and exercise. (2015) 47:1372-82.doi:10.1249/MSS.0000000000000540

48. Temesi J, Besson T, Parent A, Singh B, Martin V, Brownstein CG, et al. Effect of race distance on performance fatigability in male trail and ultra-trail runners. SCANDINAVIAN JOURNAL OF MEDICINE & SCIENCE IN SPORTS. (2021) 31:1809-21.doi:10.1111/sms.14004

49. Temesi J, Rupp T, Martin V, Arnal PJ, Feasson L, Verges S, et al. Central Fatigue Assessed by Transcranial Magnetic Stimulation in Ultratrail Running. Medicine and science in sports and exercise. (2014) 46:1166-75.doi:10.1249/MSS.0000000000000207

50. Tomazin K, Millet GY, Ulaga M, Jereb B, Strojnik V. Peripheral alterations after two different concentric power protocols. EUROPEAN JOURNAL OF SPORT SCIENCE. (2011) 11:327-33.doi:10.1080/17461391.2010.521582

51. Vernillo G, Khassetarash A, Millet GY, Temesi J. Use of transcranial magnetic stimulation to assess relaxation rates in unfatigued and fatigued knee-extensor muscles. EXPERIMENTAL BRAIN RESEARCH. (2021) 239:205-16.doi:10.1007/s00221-020-05921-9

52. Behm DG, Peach A, Maddigan M, Aboodarda SJ, DiSanto MC, Button DC, et al. Massage and stretching reduce spinal reflex excitability without affecting twitch contractile properties. JOURNAL OF ELECTROMYOGRAPHY AND KINESIOLOGY. (2013) 23:1215-21.doi:10.1016/j.jelekin.2013.05.002

53. Botter A, Oprandi G, Lanfranco F, Allasia S, Maffiuletti NA, Minetto MA. Atlas of the muscle motor points for the lower limb: implications for electrical stimulation procedures and electrode positioning. European journal of applied physiology. (2011) 111:2461-71.doi:10.1007/s00421-011-2093-y

54. Carbonaro M, Seynnes O, Maffiuletti NA, Busso C, Minetto MA, Botter A. Architectural Changes in Superficial and Deep Compartments of the Tibialis Anterior During Electrical Stimulation Over Different Sites. IEEE TRANSACTIONS ON NEURAL SYSTEMS AND REHABILITATION ENGINEERING. (2020) 28:2557-65.doi:10.1109/TNSRE.2020.3027037

55. Casartelli NC, Item-Glatthorn JF, Friesenbichler B, Bizzini M, Salzmann GM, Maffiuletti NA. Quadriceps Neuromuscular Impairments after Arthroscopic Knee Surgery: Comparison between Procedures. JOURNAL OF CLINICAL MEDICINE. (2019) 8.doi:10.3390/jcm8111881

56. Cattagni T, Lepers R, Maffiuletti NA. Effects of neuromuscular electrical stimulation on contralateral quadriceps function. JOURNAL OF ELECTROMYOGRAPHY AND KINESIOLOGY. (2018) 38:111-8.doi:10.1016/j.jelekin.2017.11.013

57. Crivelli G, Borrani F, Capt R, Gremion G, Maffiuletti NA. Actions of beta(2)-Adrenoceptor Agonist Drug on Human Soleus Muscle Contraction. Medicine and science in sports and exercise. (2013) 45:1252-60.doi:10.1249/MSS.0b013e318284706a

58. Crivelli G, Maffiuletti NA. Actions of beta(2)-Adrenoceptor Agonist Drug on Neuromuscular Function after Fatigue. Medicine and science in sports and exercise. (2014) 46:247-56.doi:10.1249/MSS.0b013e3182a54ee3

59. Da Silva SRD, Neyroud D, Maffiuletti NA, Gondin J, Place N. TWITCH POTENTIATION INDUCED BY TWO DIFFERENT MODALITIES OF NEUROMUSCULAR ELECTRICAL STIMULATION: IMPLICATIONS FOR MOTOR UNIT RECRUITMENT. Muscle & nerve. (2015) 51:412-8.doi:10.1002/mus.24315

60. Giroux C, Roduit B, Rodriguez-Falces J, Duchateau J, Maffiuletti NA, Place N. Short vs. long pulses for testing knee extensor neuromuscular properties: does it matter? European journal of applied physiology. (2018) 118:361-9.doi:10.1007/s00421-017-3778-7

61. Gondin J, Brocca L, Bellinzona E, D'Antona G, Maffiuletti NA, Miotti D, et al. Neuromuscular electrical stimulation training induces atypical adaptations of the human skeletal muscle phenotype: a functional and proteomic analysis. JOURNAL OF APPLIED PHYSIOLOGY. (2011) 110:433-50.doi:10.1152/japplphysiol.00914.2010

62. Herzig D, Maffiuletti NA, Eser P. The Application of Neuromuscular Electrical Stimulation Training in Various Non-neurologic Patient Populations: A Narrative Review. PM&R. (2015) 7:1167-78.doi:10.1016/j.pmrj.2015.03.022

63. Hortobagyi T, Maffiuletti NA. Neural adaptations to electrical stimulation strength training. European journal of applied physiology. (2011) 111:2439-49.doi:10.1007/s00421-011-2012-2

64. Maffiuletti NA, Green DA, Vaz MA, Dirks ML. Neuromuscular Electrical Stimulation as a Potential Countermeasure for Skeletal Muscle Atrophy and Weakness During Human Spaceflight. FRONTIERS IN PHYSIOLOGY. (2019) 10.doi:10.3389/fphys.2019.01031

65. Maffiuletti NA, Morelli A, Martin A, Duclay J, Billot M, Jubeau M, et al. EFFECT OF GENDER AND OBESITY ON ELECTRICAL CURRENT THRESHOLDS. Muscle & nerve. (2011) 44:202-7.doi:10.1002/mus.22050

66. Maffiuletti NA, Roig M, Karatzanos E, Nanas S. Neuromuscular electrical stimulation for preventing skeletal-muscle weakness and wasting in critically ill patients: a systematic review. BMC MEDICINE. (2013) 11.doi:10.1186/1741-7015-11-137

67. Maffiuletti NA, Vivodtzev I, Minetto MA, Place N. A new paradigm of neuromuscular electrical stimulation for the quadriceps femoris muscle. European journal of applied physiology. (2014) 114:1197-205.doi:10.1007/s00421-014-2849-2

68. Medeiros FVA, Vieira A, Carregaro RL, Bottaro M, Maffiuletti NA, Durigan JLQ. Skinfold thickness affects the isometric knee extension torque evoked by Neuromuscular Electrical Stimulation. BRAZILIAN JOURNAL OF PHYSICAL THERAPY. (2015) 19:466-72.doi:10.1590/bjpt-rbf.2014.0114

69. Minetto MA, Botter A, Gamerro G, Varvello I, Massazza G, Bellomo RG, et al. Contralateral effect of short-duration unilateral neuromuscular electrical stimulation and focal vibration in healthy subjects. EUROPEAN JOURNAL OF PHYSICAL AND REHABILITATION MEDICINE. (2018) 54:911-20.doi:10.23736/S1973-9087.18.05004-9

70. Morf C, Lauer VW, Casartelli NC, Maffiuletti NA. Acute Effects of Multipath Electrical Stimulation in Patients With Total Knee Arthroplasty. Archives of physical medicine and rehabilitation. (2015) 96:498-504.doi:10.1016/j.apmr.2014.10.011

71. Neyroud D, Dodd D, Gondin J, Maffiuletti NA, Kayser B, Place N. Wide-pulse-high-frequency neuromuscular stimulation of triceps surae induces greater muscle fatigue compared with conventional stimulation. JOURNAL OF APPLIED PHYSIOLOGY. (2014) 116:1281-9.doi:10.1152/japplphysiol.01015.2013

72. Neyroud D, Gonzalez M, Mueller S, Agostino D, Grospretre S, Maffiuletti NA, et al. Neuromuscular adaptations to wide-pulse high-frequency neuromuscular electrical stimulation training. European journal of applied physiology. (2019) 119:1105-16.doi:10.1007/s00421-019-04100-1

73. Neyroud D, Maffiuletti NA, Kayser B, Place N. Mechanisms of Fatigue and Task Failure Induced By Sustained Submaximal Contractions. Medicine and science in sports and exercise. (2012) 44:1243-51.doi:10.1249/MSS.0b013e318245cc4d

74. Rabello R, Frohlich M, Maffiuletti NA, Vaz MA. Influence of Pulse Waveform and Frequency on Evoked Torque, Stimulation Efficiency, and Discomfort in Healthy Subjects. American journal of physical medicine & rehabilitation. (2021) 100:161-7.doi:10.1097/PHM.0000000000001541

75. Rodriguez-Falces J, Maffiuletti NA, Place N. TWITCH AND M-WAVE POTENTIATION INDUCED BY INTERMITTENT MAXIMAL VOLUNTARY QUADRICEPS CONTRACTIONS: DIFFERENCES BETWEEN DIRECT QUADRICEPS AND FEMORAL NERVE STIMULATION. Muscle & nerve. (2013) 48:920-9.doi:10.1002/mus.23856

76. Rodriguez-Falces J, Maffiuletti NA, Place N. SPATIAL DISTRIBUTION OF MOTOR UNITS RECRUITED DURING ELECTRICAL STIMULATION OF THE QUADRICEPS MUSCLE VERSUS THE FEMORAL NERVE. Muscle & nerve. (2013) 48:752-61.doi:10.1002/mus.23811

77. Seyri KM, Maffiuletti NA. Effect of Electromyostimulation Training on Muscle Strength and Sports Performance. STRENGTH AND CONDITIONING JOURNAL. (2011) 33:70-5.doi:10.1519/SSC.0b013e3182079f11

78. Spector P, Laufer Y, Gabyzon ME, Kittelson A, Lapsley JS, Maffiuletti NA. Neuromuscular Electrical Stimulation Therapy to Restore Quadriceps Muscle Function in Patients After Orthopaedic Surgery A Novel Structured Approach. JOURNAL OF BONE AND JOINT SURGERY-AMERICAN VOLUME. (2016) 98:2017-24.doi:10.2106/JBJS.16.00192

79. Vaz MA, Frohlich M, da Silva DP, Schildt A, Thome PRO, Muller AF, et al. Development and reliability of a new system for bedside evaluation of non-volitional knee extension force. MEDICAL ENGINEERING & PHYSICS. (2021) 98:28-35.doi:10.1016/j.medengphy.2021.10.007

80. Veldman MP, Gondin J, Place N, Maffiuletti NA. Effects of Neuromuscular Electrical Stimulation Training on Endurance Performance. FRONTIERS IN PHYSIOLOGY. (2016) 7.doi:10.3389/fphys.2016.00544

81. Visscher RMS, Rossi D, Friesenbichler B, Dohm-Acker M, Rosenheck T, Maffiuletti NA. VASTUS MEDIALIS AND LATERALIS ACTIVITY DURING VOLUNTARY AND STIMULATED CONTRACTIONS. Muscle & nerve. (2017) 56:968-74.doi:10.1002/mus.25542

82. Wegrzyk J, Foure A, Le Fur Y, Maffiuletti NA, Vilmen C, Guye M, et al. Responders to Wide-Pulse, High-Frequency Neuromuscular Electrical Stimulation Show Reduced Metabolic Demand: A P-31-MRS Study in Humans. PLOS ONE. (2015) 10.doi:10.1371/journal.pone.0143972

83. Wegrzyk J, Foure A, Vilmen C, Ghattas B, Maffiuletti NA, Mattei JP, et al. Extra Forces induced by wide-pulse, high-frequency electrical stimulation: Occurrence, magnitude, variability and underlying mechanisms. CLINICAL NEUROPHYSIOLOGY. (2015) 126:1400-12.doi:10.1016/j.clinph.2014.10.001

84. Wellauer V, Morf C, Minetto MA, Place N, Maffiuletti NA. ASSESSMENT OF QUADRICEPS MUSCLE INACTIVATION WITH A NEW ELECTRICAL STIMULATION PARADIGM. Muscle & nerve. (2015) 51:117-24.doi:10.1002/mus.24266

85. Zange JC, Schopen K, Albracht K, Gerlach DA, Frings-Meuthen P, Maffiuletti NA, et al. Using the Hephaistos orthotic device to study countermeasure effectiveness of neuromuscular electrical stimulation and dietary lupin protein supplementation, a randomised controlled trial. PLOS ONE. (2017) 12.doi:10.1371/journal.pone.0171562

86. Cheng AJ, Neyroud D, Kayser B, Westerblad H, Place N. Intramuscular Contributions to Low-Frequency Force Potentiation Induced by a High-Frequency Conditioning Stimulation. FRONTIERS IN PHYSIOLOGY. (2017) 8.doi:10.3389/fphys.2017.00712

87. Cheng AJ, Place N, Bruton JD, Holmberg HC, Westerblad H. Doublet discharge stimulation increases sarcoplasmic reticulum Ca2+ release and improves performance during fatiguing contractions in mouse muscle fibres. JOURNAL OF PHYSIOLOGY-LONDON. (2013) 591:3739-48.doi:10.1113/jphysiol.2013.257188

88. Deligianni X, Hirschmann A, Place N, Bieri O, Santini F. Dynamic MRI of plantar flexion: A comprehensive repeatability study of electrical stimulation-gated muscle contraction standardized on evoked force. PLOS ONE. (2020) 15.doi:10.1371/journal.pone.0241832

89. Deligianni X, Klenk C, Place N, Garcia M, Pansini M, Hirschmann A, et al. Dynamic MR imaging of the skeletal muscle in young and senior volunteers during synchronized minimal neuromuscular electrical stimulation. MAGNETIC RESONANCE MATERIALS IN PHYSICS BIOLOGY AND MEDICINE. (2020) 33:393-400.doi:10.1007/s10334-019-00787-7

90. Dittrich N, Agostino D, Philippe RA, Guglielmo LGA, Place N. Effect of hypnotic suggestion on knee extensor neuromuscular properties in resting and fatigued states. PLOS ONE. (2018) 13.doi:10.1371/journal.pone.0195437

91. Neyroud D, Armand S, De Coulon G, Da Silva SRD, Wegrzyk J, Gondin J, et al. Wide-pulse-high-frequency neuromuscular electrical stimulation in cerebral palsy. CLINICAL NEUROPHYSIOLOGY. (2016) 127:1530-9.doi:10.1016/j.clinph.2015.07.009

92. Neyroud D, Cheng AJ, Bourdillon N, Kayser B, Place N, Westerblad H. Muscle Fatigue Affects the Interpolated Twitch Technique When Assessed Using Electrically-Induced Contractions in Human and Rat Muscles. FRONTIERS IN PHYSIOLOGY. (2016) 7.doi:10.3389/fphys.2016.00252

93. Neyroud D, Cheng AJ, Donnelly C, Bourdillon N, Gassner AL, Geiser L, et al. Toxic doses of caffeine are needed to increase skeletal muscle contractility. AMERICAN JOURNAL OF PHYSIOLOGY-CELL PHYSIOLOGY. (2019) 316:C246-C51.doi:10.1152/ajpcell.00269.2018

94. Neyroud D, Samararatne J, Kayser B, Place N. Neuromuscular Fatigue After Repeated Jumping With Concomitant Electrical Stimulation. INTERNATIONAL JOURNAL OF SPORTS PHYSIOLOGY AND PERFORMANCE. (2017) 12:1335-40.doi:10.1123/ijspp.2016-0571

95. Peyrard A, Willis SJ, Place N, Millet GP, Borrani F, Rupp T. Neuromuscular evaluation of arm-cycling repeated sprints under hypoxia and/or blood flow restriction. European journal of applied physiology. (2019) 119:1533-45.doi:10.1007/s00421-019-04143-4

96. Rodriguez-Falces J, Place N. Recruitment order of quadriceps motor units: femoral nerve vs. direct quadriceps stimulation. European journal of applied physiology. (2013) 113:3069-77.doi:10.1007/s00421-013-2736-2

97. Rodriguez-Falces J, Place N. Different recoveries of the first and second phases of the M-wave after intermittent maximal voluntary contractions. European journal of applied physiology. (2017) 117:607-18.doi:10.1007/s00421-017-3553-9

98. Rodriguez-Falces J, Place N. Determinants, analysis and interpretation of the muscle compound action potential (M wave) in humans: implications for the study of muscle fatigue. European journal of applied physiology. (2018) 118:501-21.doi:10.1007/s00421-017-3788-5

99. Rodriguez-Falces J, Place N. Sarcolemmal membrane excitability during repeated intermittent maximal voluntary contractions. EXPERIMENTAL PHYSIOLOGY. (2019) 104:136-48.doi:10.1113/EP087218

100. Spring JN, Place N, Borrani F, Kayser B, Barral J. Movement-Related Cortical Potential Amplitude Reduction after Cycling Exercise Relates to the Extent of Neuromuscular Fatigue. FRONTIERS IN HUMAN NEUROSCIENCE. (2016) 10.doi:10.3389/fnhum.2016.00257

101. Abilmona SM, Gorgey AS. Associations of the trunk skeletal musculature and dietary intake to biomarkers of cardiometabolic health after spinal cord injury. Clinical physiology and functional imaging. (2018) 38:949-58.doi:10.1111/cpf.12505

102. Chandrasekaran S, Davis J, Bersch I, Goldberg G, Gorgey AS. Electrical stimulation and denervated muscles after spinal cord injury. Neural regeneration research. (2020) 15:1397-407.doi:10.4103/1673-5374.274326

103. Dolbow DR, Gorgey AS. Effects of Use and Disuse on Non-paralyzed and Paralyzed Skeletal Muscles. AGING AND DISEASE. (2016) 7.doi:10.14336/AD.2015.0826

104. Dolbow DR, Gorgey AS, Khalil RK, Gater DR. Effects of a fifty-six month electrical stimulation cycling program after tetraplegia: case report. JOURNAL OF SPINAL CORD MEDICINE. (2017) 40:485-8.doi:10.1080/10790268.2016.1234750

105. Dolbow DR, Gorgey AS, Sutor TW, Bochkezanian V, Musselman K. Invasive and Non-Invasive Approaches of Electrical Stimulation to Improve Physical Functioning after Spinal Cord Injury. JOURNAL OF CLINICAL MEDICINE. (2021) 10.doi:10.3390/jcm10225356

106. Ghatas MP, Lester RM, Khan MR, Gorgey AS. Semi-automated segmentation of magnetic resonance images for thigh skeletal muscle and fat using threshold technique after spinal cord injury. Neural regeneration research. (2018) 13:1787-95.doi:10.4103/1673-5374.238623

107. Gorgey AS, Caudill C, Khalil RE. Effects of once weekly NMES training on knee extensors fatigue and body composition in a person with spinal cord injury. JOURNAL OF SPINAL CORD MEDICINE. (2016) 39:99-102.doi:10.1179/2045772314Y.0000000293

108. Gorgey AS, Cho GM, Dolbow DR, Gater DR. Differences in current amplitude evoking leg extension in individuals with spinal cord injury. NEUROREHABILITATION. (2013) 33:161-70.doi:10.3233/NRE-130941

109. Gorgey AS, Dolbow DR, Cifu DX, Gater DR. Neuromuscular electrical stimulation attenuates thigh skeletal muscles atrophy but not trunk muscles after spinal cord injury. JOURNAL OF ELECTROMYOGRAPHY AND KINESIOLOGY. (2013) 23:977-84.doi:10.1016/j.jelekin.2013.04.007

110. Gorgey AS, Dolbow DR, Dolbow JD, Khalil RK, Gater DR. The effects of electrical stimulation on body composition and metabolic profile after spinal cord injury - Part II. JOURNAL OF SPINAL CORD MEDICINE. (2015) 38:23-37.doi:10.1179/2045772314Y.0000000244

111. Gorgey AS, Graham ZA, Bauman WA, Cardozo C, Gater DR. Abundance in proteins expressed after functional electrical stimulation cycling or arm cycling ergometry training in persons with chronic spinal cord injury. JOURNAL OF SPINAL CORD MEDICINE. (2017) 40:439-48.doi:10.1080/10790268.2016.1229397

112. Gorgey AS, Graham ZA, Chen Q, Rivers J, Adler RA, Lesnefsky EJ, et al. Sixteen weeks of testosterone with or without evoked resistance training on protein expression, fiber hypertrophy and mitochondrial health after spinal cord injury. JOURNAL OF APPLIED PHYSIOLOGY. (2020) 128:1487-96.doi:10.1152/japplphysiol.00865.2019

113. Gorgey AS, Harnish CR, Daniels JA, Dolbow DR, Keeley A, Moore J, et al. A report of anticipated benefits of functional electrical stimulation after spinal cord injury. JOURNAL OF SPINAL CORD MEDICINE. (2012) 35:107-12.doi:10.1179/204577212X13309481546619

114. Gorgey AS, Khalil RE, Davis JC, Carter W, Gill R, Rivers J, et al. Skeletal muscle hypertrophy and attenuation of cardio-metabolic risk factors (SHARC) using functional electrical stimulation-lower extremity cycling in persons with spinal cord injury: study protocol for a randomized clinical trial. TRIALS. (2019) 20.doi:10.1186/s13063-019-3560-8

115. Gorgey AS, Khalil RE, Gill R, Gater DR, Lavis TD, Cardozo CP, et al. Low-Dose Testosterone and Evoked Resistance Exercise after Spinal Cord Injury on Cardio-Metabolic Risk Factors: An Open-Label Randomized Clinical Trial. JOURNAL OF NEUROTRAUMA. (2019) 36:2631-45.doi:10.1089/neu.2018.6136

116. Gorgey AS, Khalil RE, Gill R, O'Brien LC, Lavis T, Castillo T, et al. Effects of Testosterone and Evoked Resistance Exercise after Spinal Cord Injury (TEREX-SCI): study protocol for a randomised controlled trial. BMJ OPEN. (2017) 7.doi:10.1136/bmjopen-2016-014125

117. Gorgey AS, Khalil RE, Lester RM, Dudley GA, Gater DR. Paradigms of Lower Extremity Electrical Stimulation Training After Spinal Cord Injury. JOVE-JOURNAL OF VISUALIZED EXPERIMENTS. (2018).doi:10.3791/57000

118. Gorgey AS, Lai RE, Khalil RE, Rivers J, Cardozo C, Chen Q, et al. Neuromuscular electrical stimulation resistance training enhances oxygen uptake and ventilatory efficiency independent of mitochondrial complexes after spinal cord injury: a randomized clinical trial. JOURNAL OF APPLIED PHYSIOLOGY. (2021) 131:265-76.doi:10.1152/japplphysiol.01029.2020

119. Gorgey AS, Lawrence J. Acute Responses of Functional Electrical Stimulation Cycling on the Ventilation-to-CO2 Production Ratio and Substrate Utilization After Spinal Cord Injury. PM&R. (2016) 8:225-34.doi:10.1016/j.pmrj.2015.10.006

120. Gorgey AS, Mather KJ, Cupp HR, Gater DR. Effects of Resistance Training on Adiposity and Metabolism after Spinal Cord Injury. Medicine and science in sports and exercise. (2012) 44:165-74.doi:10.1249/MSS.0b013e31822672aa

121. Gorgey AS, Poarch HJ, Dolbow DR, Castillo T, Gater DR. Effect of adjusting pulse durations of functional electrical stimulation cycling on energy expenditure and fatigue after spinal cord injury. JOURNAL OF REHABILITATION RESEARCH AND DEVELOPMENT. (2014) 51:1455-67.doi:10.1682/JRRD.2014.02.0054

122. Gorgey AS, Timmons MK, Dolbow DR, Bengel J, Fugate-Laus KC, Michener LA, et al. Electrical stimulation and blood flow restriction increase wrist extensor cross-sectional area and flow meditated dilatation following spinal cord injury. European journal of applied physiology. (2016) 116:1231-44.doi:10.1007/s00421-016-3385-z

123. Gorgey AS, Timmons MK, Michener LA, Ericksen JJ, Gater DR. Intra-rater Reliability of Ultrasound Imaging of Wrist Extensor Muscles in Patients With Tetraplegia. PM&R. (2014) 6:127-33.doi:10.1016/j.pmrj.2013.08.607

124. Gorgey AS, Witt O, O'Brien L, Cardozo C, Chen Q, Lesnefsky EJ, et al. Mitochondrial health and muscle plasticity after spinal cord injury. European journal of applied physiology. (2019) 119:315-31.doi:10.1007/s00421-018-4039-0

125. Holman ME, Gorgey AS. Testosterone and Resistance Training Improve Muscle Quality in Spinal Cord Injury. Medicine and science in sports and exercise. (2019) 51:1591-8.doi:10.1249/MSS.0000000000001975

126. Moore PD, Gorgey AS, Wade RC, Khalil RE, Lavis TD, Khan R, et al. Neuromuscular electrical stimulation and testosterone did not influence heterotopic ossification size after spinal cord injury: A case series. WORLD JOURNAL OF CLINICAL CASES. (2016) 4:172-6.doi:10.12998/wjcc.v4.i7.172

127. O'Brien LC, Chen Q, Savas J, Lesnefsky EJ, Gorgey AS. Skeletal muscle mitochondrial mass is linked to lipid and metabolic profile in individuals with spinal cord injury. European journal of applied physiology. (2017) 117:2137-47.doi:10.1007/s00421-017-3687-9

128. Wade RC, Lester RM, Gorgey AS. Validation of Anthropometric Muscle Cross-Sectional Area Equation after Spinal Cord Injury. INTERNATIONAL JOURNAL OF SPORTS MEDICINE. (2018) 39:366-73.doi:10.1055/s-0044-102133

129. Foure A, Duhamel G, Wegrzyk J, Boudinet H, Mattei JP, Le Troter A, et al. Heterogeneity of Muscle Damage Induced by Electrostimulation: A Multimodal MRI Study. Medicine and science in sports and exercise. (2015) 47:166-75.doi:10.1249/MSS.0000000000000397

130. Foure A, Gondin J. Skeletal Muscle Damage Produced by Electrically Evoked Muscle Contractions. Exercise and sport sciences reviews. (2021) 49:59-65.doi:10.1249/JES.0000000000000239

131. Foure A, Le Troter A, Ogler AC, Guye M, Gondin J, Bendahan D. Spatial difference can occur between activated and damaged muscle areas following electrically-induced isometric contractions. JOURNAL OF PHYSIOLOGY-LONDON. (2019) 597:4227-36.doi:10.1113/JP278205

132. Foure A, Nosaka K, Wegrzyk J, Duhamel G, Le Troter A, Boudinet H, et al. Time Course of Central and Peripheral Alterations after Isometric Neuromuscular Electrical Stimulation-Induced Muscle Damage. PLOS ONE. (2014) 9.doi:10.1371/journal.pone.0107298

133. Foure A, Ogier AC, Guye M, Gondin J, Bendahan D. Muscle alterations induced by electrostimulation are lower at short quadriceps femoris length. European journal of applied physiology. (2020) 120:325-35.doi:10.1007/s00421-019-04277-5

134. Foure A, Wegrzyk J, Le Fur Y, Mattei JP, Boudinet H, Vilmen C, et al. Impaired Mitochondrial Function and Reduced Energy Cost as a Result of Muscle Damage. Medicine and science in sports and exercise. (2015) 47:1135-44.doi:10.1249/MSS.0000000000000523

135. Gondin J, Cozzone PJ, Bendahan D. Is high-frequency neuromuscular electrical stimulation a suitable tool for muscle performance improvement in both healthy humans and athletes? European journal of applied physiology. (2011) 111:2473-87.doi:10.1007/s00421-011-2101-2

136. Gondin J, Giannesini B, Vilmen C, Le Fur Y, Cozzone PJ, Bendahan D. Effects of a single bout of isometric neuromuscular electrical stimulation on rat gastrocnemius muscle: A combined functional, biochemical and MRI investigation. JOURNAL OF ELECTROMYOGRAPHY AND KINESIOLOGY. (2011) 21:525-32.doi:10.1016/j.jelekin.2011.01.006

137. Gondin J, Vilmen C, Cozzone PJ, Bendahan D, Duhamel G. High-field (11.75T) multimodal MR imaging of exercising hindlimb mouse muscles using a non-invasive combined stimulation and force measurement device. NMR IN BIOMEDICINE. (2014) 27:870-9.doi:10.1002/nbm.3122

138. Hilmi M, Jouinot A, Burns R, Pigneur F, Mounier R, Gondin J, et al. Body composition and sarcopenia: The next-generation of personalized oncology and pharmacology? PHARMACOLOGY & THERAPEUTICS. (2019) 196:135-59.doi:10.1016/j.pharmthera.2018.12.003

139. Jubeau M, Le Fur Y, Duhamel G, Wegrzyk J, Confort-Gouny S, Vilmen C, et al. Localized Metabolic and T-2 Changes Induced by Voluntary and Evoked Contractions. Medicine and science in sports and exercise. (2015) 47:921-30.doi:10.1249/MSS.0000000000000491

140. Martin A, Grospretre S, Vilmen C, Guye M, Mattei JP, Le Fur Y, et al. The Etiology of Muscle Fatigue Differs between Two Electrical Stimulation Protocols. Medicine and science in sports and exercise. (2016) 48:1474-84.doi:10.1249/MSS.0000000000000930

141. Porcelli S, Marzorati M, Pugliese L, Adamo S, Gondin J, Bottinelli R, et al. Lack of functional effects of neuromuscular electrical stimulation on skeletal muscle oxidative metabolism in healthy humans. JOURNAL OF APPLIED PHYSIOLOGY. (2012) 113:1101-9.doi:10.1152/japplphysiol.01627.2011

142. Rahmati M, Gondin J, Malakoutinia F. Effects of Neuromuscular Electrical Stimulation on Quadriceps Muscle Strength and Mass in Healthy Young and Older Adults: A Scoping Review. Physical therapy. (2021) 101.doi:10.1093/ptj/pzab144

143. Ato S, Tsushima D, Isono Y, Suginohara T, Maruyama Y, Nakazato K, et al. The Effect of Changing the Contraction Mode During Resistance Training on mTORC1 Signaling and Muscle Protein Synthesis. FRONTIERS IN PHYSIOLOGY. (2019) 10.doi:10.3389/fphys.2019.00406

144. Kotani T, Takegaki J, Takagi R, Nakazato K, Ishii N. Consecutive bouts of electrical stimulation-induced contractions alter ribosome biogenesis in rat skeletal muscle. JOURNAL OF APPLIED PHYSIOLOGY. (2019) 126:1673-80.doi:10.1152/japplphysiol.00665.2018

145. Kotani T, Takegaki J, Tamura Y, Kouzaki K, Nakazato K, Ishii N. Repeated bouts of resistance exercise in rats alter mechanistic target of rapamycin complex 1 activity and ribosomal capacity but not muscle protein synthesis. EXPERIMENTAL PHYSIOLOGY. (2021) 106:1950-60.doi:10.1113/EP089699

146. Kouzaki K, Nosaka K, Ochi E, Nakazato K. Increases in M-wave latency of biceps brachii after elbow flexor eccentric contractions in women. European journal of applied physiology. (2016) 116:939-46.doi:10.1007/s00421-016-3358-2

147. Lee K, Kouzaki K, Ochi E, Kobayashi K, Tsutaki A, Hiranuma K, et al. Eccentric contractions of gastrocnemius muscle-induced nerve damage in rats. Muscle & nerve. (2014) 50:87-94.doi:10.1002/mus.24120

148. Lee K, Ochi E, Song H, Nakazato K. Activation of AMP-activated protein kinase induce expression of FoxO1, FoxO3a, and myostatin after exercise-induced muscle damage. BIOCHEMICAL AND BIOPHYSICAL RESEARCH COMMUNICATIONS. (2015) 466:289-94.doi:10.1016/j.bbrc.2015.08.126

149. Maekawa T, Ogasawara R, Tsutaki A, Lee K, Nakada S, Nakazato K, et al. Electrically evoked local muscle contractions cause an increase in hippocampal BDNF. APPLIED PHYSIOLOGY NUTRITION AND METABOLISM. (2018) 43:491-6.doi:10.1139/apnm-2017-0536

150. Makanae Y, Ogasawara R, Sato K, Takamura Y, Matsutani K, Kido K, et al. Acute bout of resistance exercise increases vitamin D receptor protein expression in rat skeletal muscle. EXPERIMENTAL PHYSIOLOGY. (2015) 100:1168-76.doi:10.1113/EP085207

151. Mizunoya W, Miyahara H, Okamoto S, Akahoshi M, Suzuki T, Do MKQ, et al. Improvement of Endurance Based on Muscle Fiber-Type Composition by Treatment with Dietary Apple Polyphenols in Rats. PLOS ONE. (2015) 10.doi:10.1371/journal.pone.0134303

152. Mori T, Ato S, Knudsen JR, Henriquez-Olguin C, Li ZC, Wakabayashi K, et al. c-Myc overexpression increases ribosome biogenesis and protein synthesis independent of mTORC1 activation in mouse skeletal muscle. AMERICAN JOURNAL OF PHYSIOLOGY-ENDOCRINOLOGY AND METABOLISM. (2021) 321:E551-E9.doi:10.1152/ajpendo.00164.2021

153. Ochi E, Nakazato K, Ishii N. MUSCULAR HYPERTROPHY AND CHANGES IN CYTOKINE PRODUCTION AFTER ECCENTRIC TRAINING IN THE RAT SKELETAL MUSCLE. Journal of strength and conditioning research. (2011) 25:2283-92.doi:10.1519/JSC.0b013e3181f1592e

154. Ochi E, Nosaka K, Tsutaki A, Kouzaki K, Nakazato K. Repeated bouts of fast velocity eccentric contractions induce atrophy of gastrocnemius muscle in rats. JOURNAL OF MUSCLE RESEARCH AND CELL MOTILITY. (2015) 36:317-27.doi:10.1007/s10974-015-9426-0

155. Ochi E, Ueda H, Tsuchiya Y, Kouzaki K, Nakazato K. Eccentric contraction-induced muscle damage in human flexor pollicis brevis is accompanied by impairment of motor nerve. SCANDINAVIAN JOURNAL OF MEDICINE & SCIENCE IN SPORTS. (2020) 30:462-71.doi:10.1111/sms.13589

156. Ogasawara R, Kobayashi K, Tsutaki A, Lee K, Abe T, Fujita S, et al. mTOR signaling response to resistance exercise is altered by chronic resistance training and detraining in skeletal muscle. JOURNAL OF APPLIED PHYSIOLOGY. (2013) 114:934-40.doi:10.1152/japplphysiol.01161.2012

157. Ogasawara R, Sato K, Higashida K, Nakazato K, Fujita S. Ursolic acid stimulates mTORC1 signaling after resistance exercise in rat skeletal muscle. AMERICAN JOURNAL OF PHYSIOLOGY-ENDOCRINOLOGY AND METABOLISM. (2013) 305:E760-E5.doi:10.1152/ajpendo.00302.2013

158. Ogasawara R, Sato K, Matsutani K, Nakazato K, Fujita S. The order of concurrent endurance and resistance exercise modifies mTOR signaling and protein synthesis in rat skeletal muscle. AMERICAN JOURNAL OF PHYSIOLOGY-ENDOCRINOLOGY AND METABOLISM. (2014) 306:E1155-E62.doi:10.1152/ajpendo.00647.2013

159. Saito K, Tamaki T, Hirata M, Hashimoto H, Nakazato K, Nakajima N, et al. Reconstruction of Multiple Facial Nerve Branches Using Skeletal Muscle-Derived Multipotent Stem Cell Sheet-Pellet Transplantation. PLOS ONE. (2015) 10.doi:10.1371/journal.pone.0138371

160. Sumi K, Ashida K, Nakazato K. Resistance exercise with anti-inflammatory foods attenuates skeletal muscle atrophy induced by chronic inflammation. JOURNAL OF APPLIED PHYSIOLOGY. (2020) 128:197-211.doi:10.1152/japplphysiol.00585.2019

161. Tamura Y, Kouzaki K, Kotani T, Nakazato K. Electrically stimulated contractile activity-induced transcriptomic responses and metabolic remodeling in C2C12 myotubes: twitch vs. tetanic contractions. AMERICAN JOURNAL OF PHYSIOLOGY-CELL PHYSIOLOGY. (2020) 319:C1029-C44.doi:10.1152/ajpcell.00494.2019

162. Tsutaki A, Ogasawara R, Kobayashi K, Lee K, Kouzaki K, Nakazato K. Effect of Intermittent Low-Frequency Electrical Stimulation on the Rat Gastrocnemius Muscle. BIOMED RESEARCH INTERNATIONAL. (2013) 2013.doi:10.1155/2013/480620
